# FOCAL3D: A 3-dimensional clustering package for single-molecule localization microscopy

**DOI:** 10.1371/journal.pcbi.1008479

**Published:** 2020-12-08

**Authors:** Daniel F. Nino, Daniel Djayakarsana, Joshua N. Milstein

**Affiliations:** 1 Department of Physics, University of Toronto, Toronto, Ontario, Canada; 2 Department of Chemical and Physical Sciences, University of Toronto Mississauga, Mississauga, Ontario, Canada; University of Virginia, UNITED STATES

## Abstract

Single-molecule localization microscopy (SMLM) is a powerful tool for studying intracellular structure and macromolecular organization at the nanoscale. The increasingly massive pointillistic data sets generated by SMLM require the development of new and highly efficient quantification tools. Here we present FOCAL3D, an accurate, flexible and exceedingly fast (scaling linearly with the number of localizations) density-based algorithm for quantifying spatial clustering in large 3D SMLM data sets. Unlike DBSCAN, which is perhaps the most commonly employed density-based clustering algorithm, an optimum set of parameters for FOCAL3D may be objectively determined. We initially validate the performance of FOCAL3D on simulated datasets at varying noise levels and for a range of cluster sizes. These simulated datasets are used to illustrate the parametric insensitivity of the algorithm, in contrast to DBSCAN, and clustering metrics such as the F1 and Silhouette score indicate that FOCAL3D is highly accurate, even in the presence of significant background noise and mixed populations of variable sized clusters, once optimized. We then apply FOCAL3D to 3D astigmatic dSTORM images of the nuclear pore complex (NPC) in human osteosaracoma cells, illustrating both the validity of the parameter optimization and the ability of the algorithm to accurately cluster complex, heterogeneous 3D clusters in a biological dataset. FOCAL3D is provided as an open source software package written in Python.

## Author summary

We have developed an accurate, highly-efficient and flexible algorithm for quantifying spatial clustering in large, 3-dimensional single-molecule localization microscopy (SMLM) datasets. Our method, FOCAL3D, is provided as an open-source software package written in Python. FOCAL3D scales linearly with the number of localizations and the algorithmic parameters may be systematically optimized so that the resulting analysis is insensitive to variation over a range of parameter choices. We initially validate the performance and parametric insensitivity of FOCAL3D on simulated datasets, then apply the algorithm to 3-dimensional, astigmatic dSTORM images of the nuclear pore complex in human osteosarcoma cells.

This is a *PLOS Computational Biology* Methods paper.

## Introduction

Single-molecule localization microscopy (SMLM) techniques [1] such as photo-activated localization microscopy (PALM) [2], direct stochastic optical reconstruction microscopy (dSTORM) [3], DNA-based point accumulation for imaging in nanoscale topography (PAINT) [4], and so on, have enabled us to visualize aspects of the biological world at an unprecedented level of detail [5]. These techniques can resolve cellular features 1-2 orders of magnitude smaller than what is achievable by conventional, diffraction-limited light microscopy [6]. Typical lateral resolutions are approximately 10-20 nm with slightly poorer depth resolution, although recent advances suggest this may be further reduced by another factor of 10 [7, 8]. Considering that nucleic acids such as DNA or RNA are 2 nm wide and that a typical protein is on the order of 4 − 6 nm in diameter, the domain of light microscopy will soon begin to overlap with that of electron microscopy, enabling us to quantify cellular organization and physiology down to the scale of single macromolecules.

Images acquired by conventional fluorescence microscopy are diffraction-limited, spatial maps of fluorophore intensity while SMLM yields a pointillistic set of approximate single-molecule coordinates (i.e. localizations). There are various approaches to generating image reconstructions from SMLM datasets that appear to produce images analogous to conventional microscopy, albeit with an enhanced resolution. For instance, one can render a Gaussian with a width of the localization precision at each localization coordinate, but the resulting image is not actually an intensity map, rather it represents the probability density of finding a single-molecule at a given location. Regardless, image processing techniques used to analyze conventional microscopy images are often applied to SMLM image reconstructions. A more quantitative approach, however, is to work directly with the table of localizations.

Various techniques exist to analyze statistical properties of pointillistic data sets and have already been applied to analyze SMLM data, such as pair-correlation analysis [9] or the Ripley’s K-function [10]. These ensemble measures are able to quantify statistical properties of the localization data such as the degree of clustering or the average size of a cluster, but do not directly identify individual clusters within a dataset. Density based spatial clustering algorithms, on the other hand, attempt to directly assign groups of localizations to a single cluster and can provide information both on the distribution of cluster size and shape as well as quantify intracellular, spatial organization.

Density based spatial clustering with noise (DBSCAN) [11] is arguably the most popular of these methods [12, 13]. How DBSCAN performs is determined by two user-defined parameters: a length-scale *ϵ* specifying the neighbourhood in which to define a local density, and a density threshold *minPts* that determines if a point is part of a cluster or not. While there are some suggestions in the literature on how best to select these parameters, such as the heuristic of setting *minPts* = *D* + 1, where *D* is the dimensionality, and searching for elbows in k-distance plots to fix *ϵ*, these suggestions often fail in practice on SMLM datasets (for a further discussion see [15]). In practice, selection of *ϵ* and *minPts* is almost always a subjective process whereby the user tunes the parameters until DBSCAN does a reasonable job identifying clusters in the data. Tuning of the parameters requires DBSCAN to be run multiple times on each dataset, a process that can be arduous for large SMLM datasets (*n*_*l*_ > 1 × 10^6^ localizations) since DBSCAN scales on average like *n*_*l*_ log *n*_*l*_ [11]. To address these issues, a variety of ‘parameter free’ clustering algorithms have recently appeared in the literature that are specifically designed for SMLM.

Originally developed for 2-dimensional datasets, Griffié et al. [15] employ Bayesian analysis to optimize cluster selection, whereas Levet et al. [16] take a geometrical approach that makes use of Voronoi tessellation. While 2-dimensional algorithms are useful for analyzing clustering in a plane, say within the cellular membrane, many biological systems require a true 3-dimensional analysis and simply clustering a projection of the data will often lead to false artefacts. In response, both of these methods have recently been extended to handle 3-dimensional datasets [17, 18]. The runtime of these algorithms, however, is quite long, requiring several hours at best to evaluate a single analysis on a reasonably large set of 3-dimensional SMLM data. Moreover, both have their drawbacks. The Bayesian approach is quite sensitive to imaging artifacts as well as the prior settings (currently limiting detection to similar sized spherical Gaussian clusters), and the Voronoi method has difficulty identifying non-isotropic or hollow, ring-like structures [19].

Here we present FOCAL3D, a 3-dimensional implementation of our previously released Fast Optimized Clustering Algorithm for Localization Microscopy (FOCAL) [14]. FOCAL3D is a density based approach similar to DBSCAN, but performed upon an appropriately discretized spatial grid. Under most conditions, this discretization greatly speeds up the algorithm, which scales linearly with the number of localizations *n*_*l*_, enabling the user to rapidly optimize and identify clusters in SMLM data, facilitating the throughput of image analysis. FOCAL3D is highly flexible and is capable of identifying heterogenous and complex, ring-like clusters. FOCAL3D is written in Python with the source code, a user’s guide and example data made available at https://github.com/MilsteinLab/FOCAL3D. Here we additionally provide a simple graphical user interface (GUI) so the software can easily be implemented by those with no computer programming experience.

An overview of this manuscript is as follows. In Section 2.1 we provide background on the FOCAL3D clustering algorithm and, in Section 2.2, discuss the procedure for optimizing the algorithmic parameters. In Section 3.1 we present SMLM simulations used to benchmark the algorithm and, in Section 3.2, evaluate the performance of FOCAL3D in clustering the simulated data at moderate and high noise conditions as well as when the cluster populations are mixed or heterogeneous. Then, in Section 3.3, we assess the clustering of FOCAL3D applied to 3D SMLM images of the nuclear pore complex (NPC). We conclude with a discussion of our results in Section 4.

## Materials and methods

### FOCAL3D algorithm

**Fig 1:**
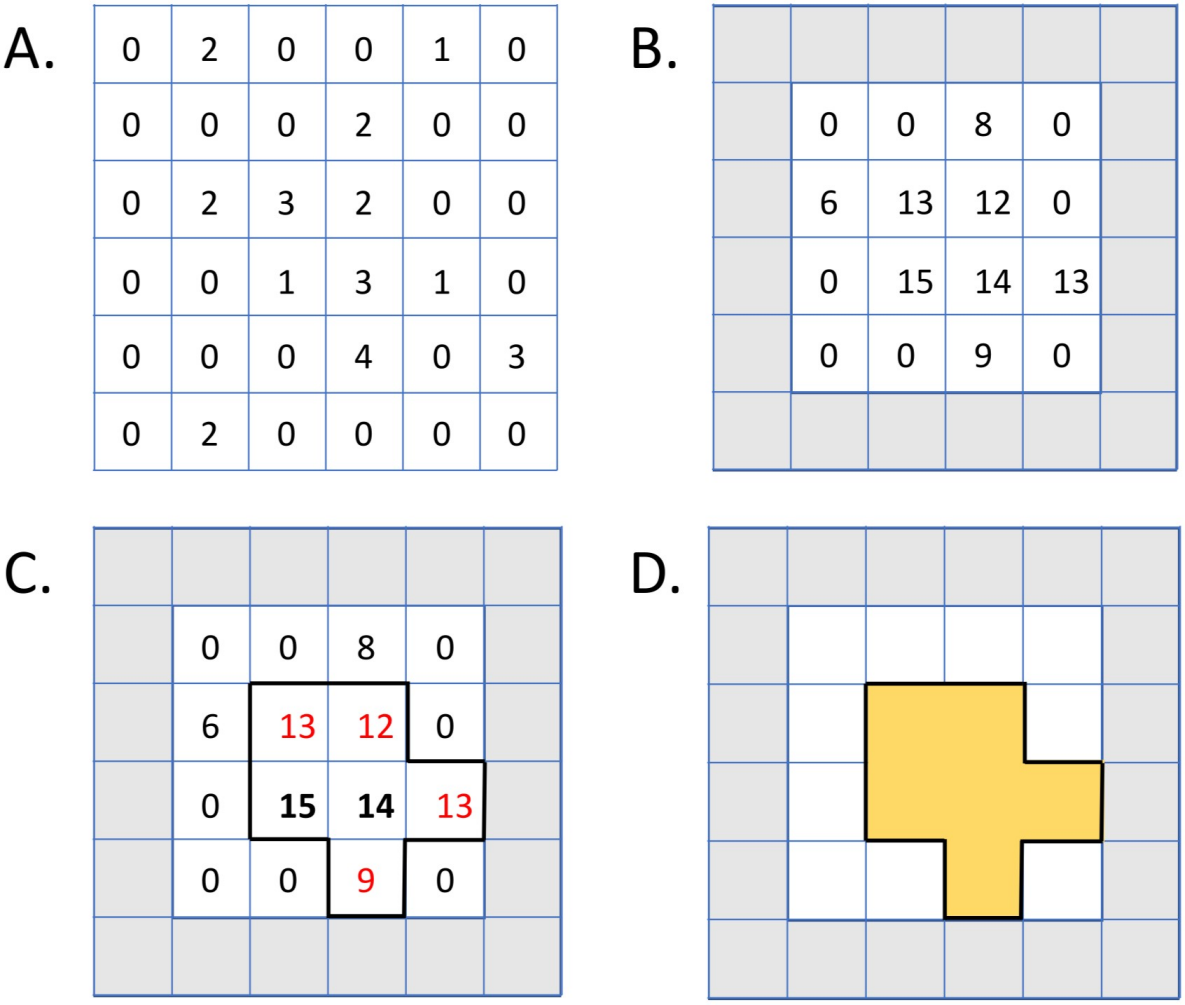
FOCAL(3D) algorithm. A.) The area (volume) is first discretized into a grid of symmetric bins with sides of length Δ, here shown in 2D, with the total number of localizations per bin indicated. B.) An enhanced density map is created by replacing each non-zero bin assignment with the sum of all neighbouring 3x3 (3x3x3) bins in 2D (3D). Bins at the edge of the sample volume are exempt from the clustering analysis as indicated by the grey boxes. C.) All voxels that exceed the threshold *minL* = 14, in this example, are categorized as core (bold) while those below the threshold that share an edge or face with a core voxel are categorized as edge voxels (red). Together, the connected core and edge voxels form candidate clusters. D.) Candidates consisting of at least *minC* connected voxels are considered as clusters (here *minC* < 6).

FOCAL3D follows a similar algorithm to the 2-dimensional implementation elaborated in [14]. Starting from a localization table, which is a list of the approximate spatial coordinates (or localizations) of all fluorescent labels detected within a sample, each localization is first assigned to a discrete spatial bin (voxel) of volume Δ^3^. This creates a discretized localization density map (Fig 1A). To enhance contrast between the noise and target (i.e. cluster) points, rather than use the raw density map, we assemble an enhanced density map by replacing the value at each voxel by a sum over all neighbouring voxels (i.e. a 3x3 grid in 2D or a 3x3x3 cubic grid in 3D centred on the voxel under consideration). This contrast enhancement is performed only for bins that contain at least one localization (Fig 1B).

Candidate clusters are selected by first identifying voxels in the enhanced density map that have a value above a density threshold *minL*. These voxels are then labeled as core voxels. Voxels adjacent to any one of the faces of a core voxel, but that did not satisfy the density threshold, are retained as well and classified as border voxels. These core and border voxels comprise the candidate clusters (Fig 1C). As we will see empirically, this way of building the clusters by connecting voxels enables the algorithm to be significantly more flexible at identifying heterogeneous and complex shaped clusters than by imposing a radius, for instance, to define the cluster volume. The final step then is to narrow down the field of candidates by imposing a threshold *minC* on cluster size, where *minC* is simply the minimum number of connected voxels containing core and border voxels that will be accepted as a cluster (Fig 1D).

A number of improvements to our original 2D MATLAB implementation of this algorithm [14] had to be incorporated in the Python implementation of FOCAL3D to efficiently handle 3D datasets. Most significantly, indexing has been largely replaced by vectorized implementations for constructing the enhanced density map, identifying border voxels and determining the size of clusters. To build the enhanced density map, we now convolve the discretized density map with a 3x3x3 kernel to perform the necessary sum of localizations over neighbouring voxels. To label the border voxels, we convolve a binary map of the core voxels with a 6-connected kernel, again eliminating the need for indexing. Finally, cluster sizes in terms of the number of connected voxels are computed using the sum function from the multi-dimensional image processing module in the Scipy library.

### Parameter selection for FOCAL3D

The overall performance of FOCAL3D is dependent upon an appropriate choice of three user-defined parameters: Δ, *minL* and *minC*. The density threshold *minL* and cluster size threshold *minC* are the discrete analogs of *minPts* and *ϵ* in DBSCAN, respectively (see Table 1). By discretizing space in FOCAL3D, we greatly enhance the speed at which we can evaluate the local density and, therefore, the full clustering of a dataset. This speed up comes at a cost, namely, the introduction of an additional parameter Δ specifying the grid size.

**Table 1:** A guide to the parameters used throughout the manuscript. Units are indicated in parentheses (with *ℓ* indicating the units of length).

	Density Threshold (# of localizations)	Local Volume	Grid Spacing
DBSCAN	*minPts*	(4*π*/3)*ϵ*^3^ (*ℓ*^3^)	-
FOCAL3D	*minL*	*minC* (*voxels*)	Δ (*ℓ*)

The grid size Δ was previously chosen in [14] based on the localization precision of the SMLM data, which reflected an uncertainty inherent in the imaging method. Unfortunately, it is not obvious how to extend our original 2D optimization to 3D datasets. Imposing a grid size on the order of the localization precision results in such a fine grid that in 3D it can drastically slow down the algorithm. Even more problematic, a fundamental step in the 2D optimization was to filter out of focus localizations by tuning *minL* until the localization precision was minimized. It is not clear how to extend this to 3D data where clusters appear at various depths and where the localization precision in *x* and *y* often differs significantly from that in the *z* plane. Moreover, the localization precision must be repeatedly calculated on the filtered data, which in 2D was done via a temporal adjacent neighbour analysis [20]–an analysis that is only valid in 2D.

In response to these technical hurdles, we developed an entirely new approach to optimizing the parameter selection that works with both 2D and 3D datasets. We show that by properly tuning Δ, the performance of FOCAL3D displays regions of insensitivity to the choice of *minC*, and that within these regions one obtains a best estimate of the ideal clustering.

#### Selecting the density threshold (*minL*)

**Fig 2:**
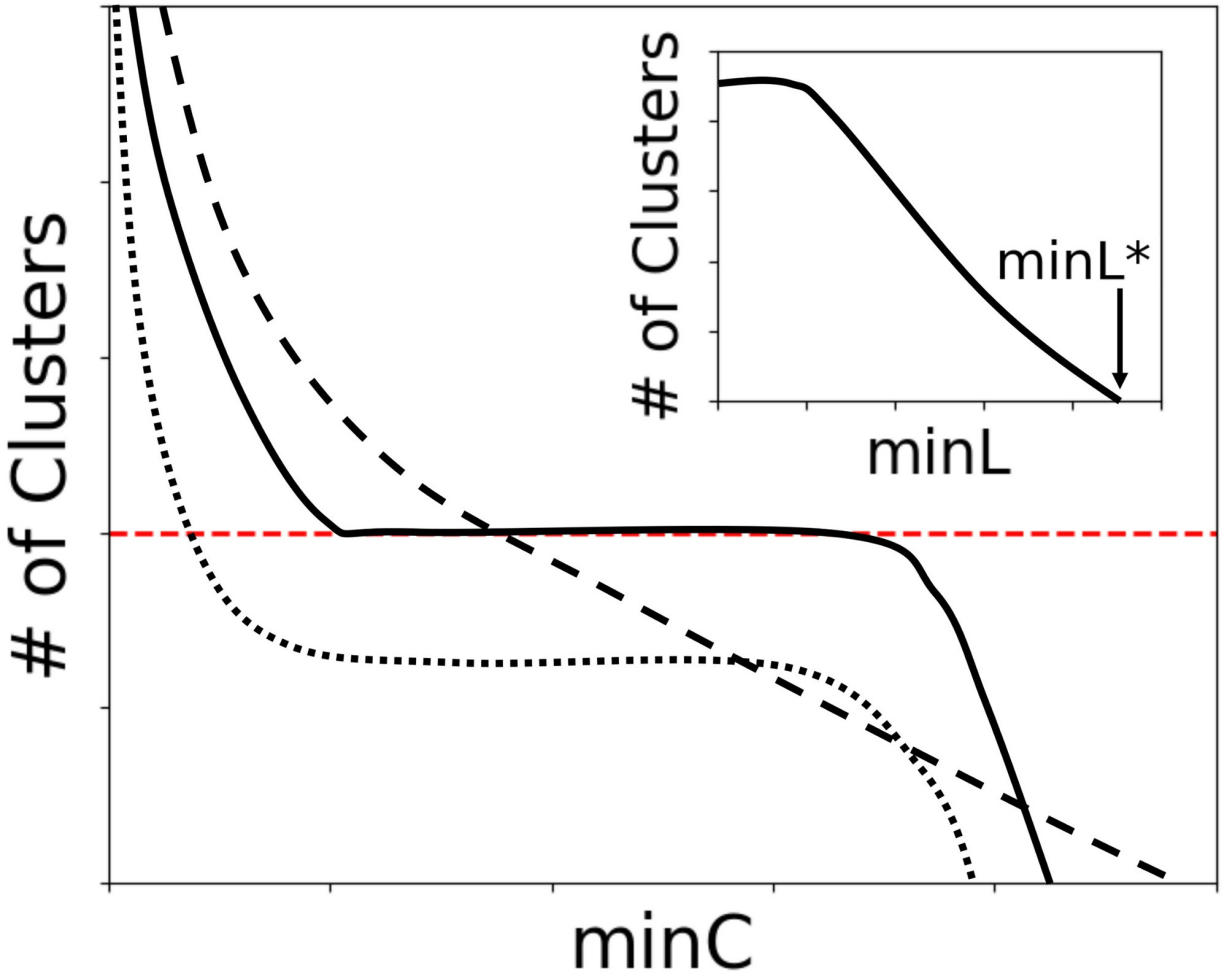
Graphical illustration of the typical behaviour seen in plots of the number of clusters vs. minC. Close to an ideal grid size Δ*, regions of insensitivity to *minC* appear (solid line). For Δ ≪ Δ*, a steep dependence on *minC* is often observed (dashed line), while for Δ ≫ Δ* a plateau in *minC* may persist, but generally yields an underestimation in the number of clusters (dotted line). The target number is indicated by the horizontal line (red dashed). Note, *minL* is tuned at each point along the curves to differentiate from a random background (inset).

For each choice of grid size Δ and cluster size threshold *minC*, it is necessary to set an optimal density threshold minL*. We first generate an artificial localization table consisting of the same number of localizations as in the actual dataset, but with each localization randomly positioned within the same volume as the experimental data. We then repeatedly run FOCAL3D on this random dataset over a range of values of *minL*, keeping Δ and *minC* constant. The optimal density threshold minL* is chosen as the lowest threshold value where FOCAL3D returns zero clusters for the randomly scattered data (see Fig 2 inset). While randomly positioned localizations may occasionally, by chance, form small clusters, these are typically filtered out by the size thresholding and have little effect on the analysis.

#### Tuning the grid size (Δ) and cluster size threshold (*minC*)

The selection of the optimum density threshold minL* can simply be automated and performed in the background at each value of the grid size Δ and the cluster size threshold *minC*. However, at present, the user must supervise the optimization of Δ and *minC*. This is done by generating a series of plots at varying Δ of the number of detected clusters vs. *minC* (now on the original localization table as opposed to a uniformly scattered set of localizations). Fig 2 illustrates the general trend of these plots for a range of grid sizes about an optimal value Δ*. For increasing Δ, near Δ* a relatively flat and extended plateau appears. At Δ* the clustering displays the largest region of insensitivity to *minC*, and along the plateau FOCAL3D is most accurate at clustering the localization data and predicting the number of clusters. For grids smaller than Δ*, the curves will typically display a monotonically decreasing trend. For grids larger than Δ*, the plateau tends to both shrink and, at values of *minC* along the plateau, the algorithm increasingly underestimates the number of clusters.

As a rule of thumb, the optimal grid size tends to fall below the cluster radius Δ* < *R*_*C*_, with too small a choice leading to an overly sensitive dependence on *minC*, and too large a choice resulting in a significant number of missed clusters. Note that it is not necessary to know the mean cluster size *R*_*C*_ *a priori*, but a good estimate can narrow the range of values for Δ that need to be searched, significantly decreasing the computational time required to optimize the algorithm. For instance, statistical models such as the Ripley’s K-function can yield a reasonable starting point for the search and can be rapidly calculated on localization data. As will be shown, there is some flexibility in the choice of grid size that will result in an accurate performance by the clustering algorithm. A user guide is provided with the software and a detailed discussion on practical aspects of implementing this optimization is given in S1 Text.

One should also note that this parameter selection routine can be translated to DBSCAN. In this case, *minPts* is found from analyzing a random distribution of the original localizations and set once the algorithm no longer detects clusters. Then, since there is no equivalent to the grid size Δ in DBSCAN, a single plot of the number of clusters detected vs. *ϵ*, optimizing *minPts* at each point, is generated and *ϵ* is set within the flat region of the curve.

## Results

### Clustering simulations

We performed a series of analyses on simulated SMLM clustering data to quantitatively assess the performance of FOCAL3D (see https://osf.io/pejaq/ for simulated datasets). Here we considered the case of 100 spherically symmetric clusters, with approximately 1 cluster per *μ*m^3^, of various mean radii: 80 ± 16 nm, 60 ± 12 nm and 40 ± 8. Like most density-based methods, FOCAL3D is not designed to deal with spatial overlap so the centroids of the simulated clusters, while otherwise randomly positioned, were well separated by at least 330 nm for the 80 nm clusters, 310 nm for the 60 nm clusters, and 290 nm for the 40 nm clusters.

Each cluster consisted of a Poisson-distributed number of ‘dyes’ randomly placed, with uniform probability, throughout the cluster volume. The mean number of dyes were ${\bar{N}}_{dyes}=20,20$, and 10 for the 80, 60, and 40 nm clusters, respectively. To account for blinking, each dye then yielded an exponentially distributed number of localizations ${\bar{N}}_{locs}=\tau_{ON}/\left(1-e^{-1/\lambda }\right)=10$ (see S1 Text), where *τ*_*ON*_ is the average ON time of a blink and λ is the characteristic number of blinks of a fluorophore. Each blink was in turn scattered about the corresponding dye centre with a Gaussian distribution whose width was sampled from a distribution of localization precisions (see S1 Text). We assumed that the spread in localization precision was slightly poorer in the axial direction (*δ*_*z*_ = 20 nm) than in the lateral plane (*δ*_*x*,*y*_ = 10 nm) reflecting the reduced axial resolution of SMLM. This also makes the underlying, symmetric clusters appear slightly elongated along the *z*-axis. The end result is clusters of an approximate mean localization density of 1 × 10^−4^ nm^−3^, 2 × 10^−4^ nm^−3^, and 4 × 10^−4^ nm^−3^ for 80, 60 and 40 nm radius clusters, respectively.

Throughout the manuscript, we quantify the performance of FOCAL3D in clustering the simulated data both in terms of precision and recall, which are standard metrics of the performance of a clustering algorithm. Precision is defined as the fraction of localizations identified as being part of a cluster that were correctly identified (*P* ≡ TruePositives/(TruePositives + FalsePositives)), while recall measures the fraction of clustered localizations identified by the algorithm (R ≡ TruePositives/(TruePositives + FalseNegatives)). By taking the weighted average of the precision and recall, these two metrics can be combined into a single measure called the *F*1 score, where *F*1 = 2*PR*/(*P* + *R*).

Another useful metric is the silhouette score *S*_*C*_, which quantifies the degree of overlap in the detected clusters. Formally, this is defined as $S_{C}=\left({\bar{d}}_{NN}-{\bar{d}}_{C}\right)/{\bar{d}}_{NN}\rangle$, where ${\bar{d}}_{NN}$ is the mean nearest-neighbour cluster distance and ${\bar{d}}_{C}$ is the mean intra-cluster distance for a sample. *S*_*C*_ will range from -1 to 1 with a higher positive score indicating more definition and spatial separation of the clusters.

### Performance clustering noisy data

We define a measure of the noise in our simulations as follows:

$$\begin{array}{c} \zeta =(\frac{{\bar{N}}_{cluster}/{\bar{V}}_{cluster}}{N_{noise}/V_{sim}}{)}^{-1}. \end{array}$$ (1)

The quantity in parentheses is the level of signal-to-noise, given here by the ratio of the mean density of clustered localizations to the density of noise. ${\bar{N}}_{cluster}$ is the mean number of localizations within a cluster, ${\bar{V}}_{cluster}$ is the mean volume of a cluster, *N*_*noise*_ is the total number of localizations uniformly added as noise to the simulation, and *V*_*sim*_ is the total simulation volume. A reasonable range of values for *ζ* to consider runs from *ζ* = 0, where there’s no noise, to *ζ* = 1 where the density of the noise is equivalent to the density of the clusters.

#### Optimization at moderate noise levels

**Fig 3:**
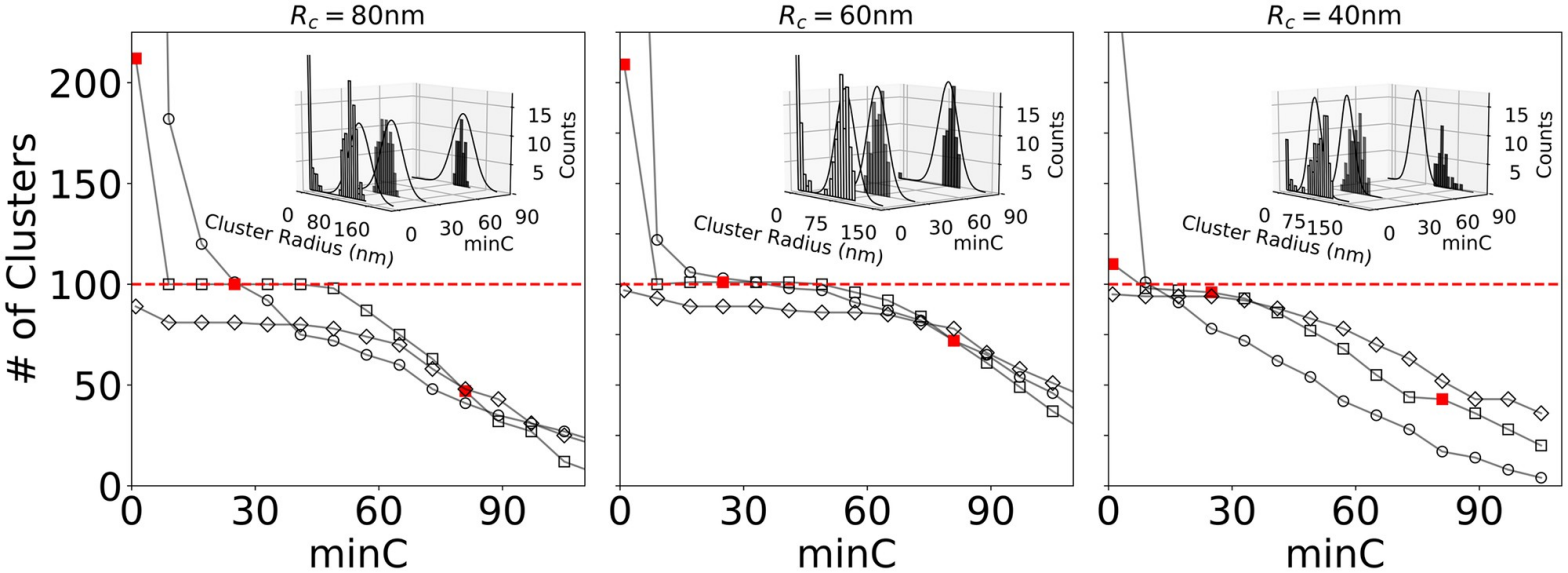
FOCAL3D performance for clusters of mean radius R_c_ = 80 nm, 60 nm and 40 nm at a noise level of *ζ* = 0.01. At each radius, the number of clusters as a function of *minC* is displayed for a range of grid sizes (*R*_*c*_ = 80 nm: Δ = 20 nm (∘), 40 nm (▫), 90 nm (◊); *R*_*c*_ = 60 nm: Δ = 20 nm (∘), 30 nm (▫), 70 nm (◊); *R*_*c*_ = 40 nm: Δ = 20 nm (∘), 40 nm (▫), 60 nm (◊)). The red, dashed line indicates the actual number of simulated clusters (100). Insets: Distribution in cluster radii estimated from convex hull determined at three points along *minC* (indicated by solid red squares). For comparison, we also display the ground truth distribution in cluster radii, shown by the solid black curves (see S1 Fig).

Fig 3 displays the number of clusters detected by FOCAL3D as a function of *minC* for a noise level of *ζ* = 0.01 and mean cluster radii of 80 nm, 60 nm and 40 nm. For 80 nm clusters, the behaviour illustrated in Fig 2 can now be seen empirically. In this case, for a grid size of half the mean cluster radius (Δ = 40 nm), the number of clusters found by FOCAL3D displays an extended plateau in the neighbourhood of the target number, providing a range of values for *minC* that yield optimal results. However, as *minC* is decreased below this optimal range, the algorithm detects an increasing number of small clusters, which leads to a severe overestimation of the number of target clusters. Likewise, as *minC* is elevated beyond the plateau, FOCAL3D begins rejecting ever larger clusters and the number of clusters predicted by the algorithm steadily decreases to zero.

If we then adjust the grid size so that too fine of a grid is chosen (Δ = 20 nm), the plateau disappears and the performance of the algorithm becomes strongly dependent on the choice of *minC* (Fig 3). While the curve does intersect with the target value, without knowing the number of clusters *a priori*, it would be challenging to select the appropriate value of *minC*. Likewise, if the grid is chosen to be too coarse (Δ = 90 nm), an insensitivity to the choice of *minC* may appear, but the algorithm tends to miss a significant amount of clusters within this region of parameter space. We should note that, to obtain the plots in Fig 3, we scanned through a range of grid sizes Δ (in 10 nm steps) and selected for our optimized choice the one that displayed the most extended plateau at the target. For clarity, not all grid sizes are shown (see S2 Fig).

**Fig 4:**
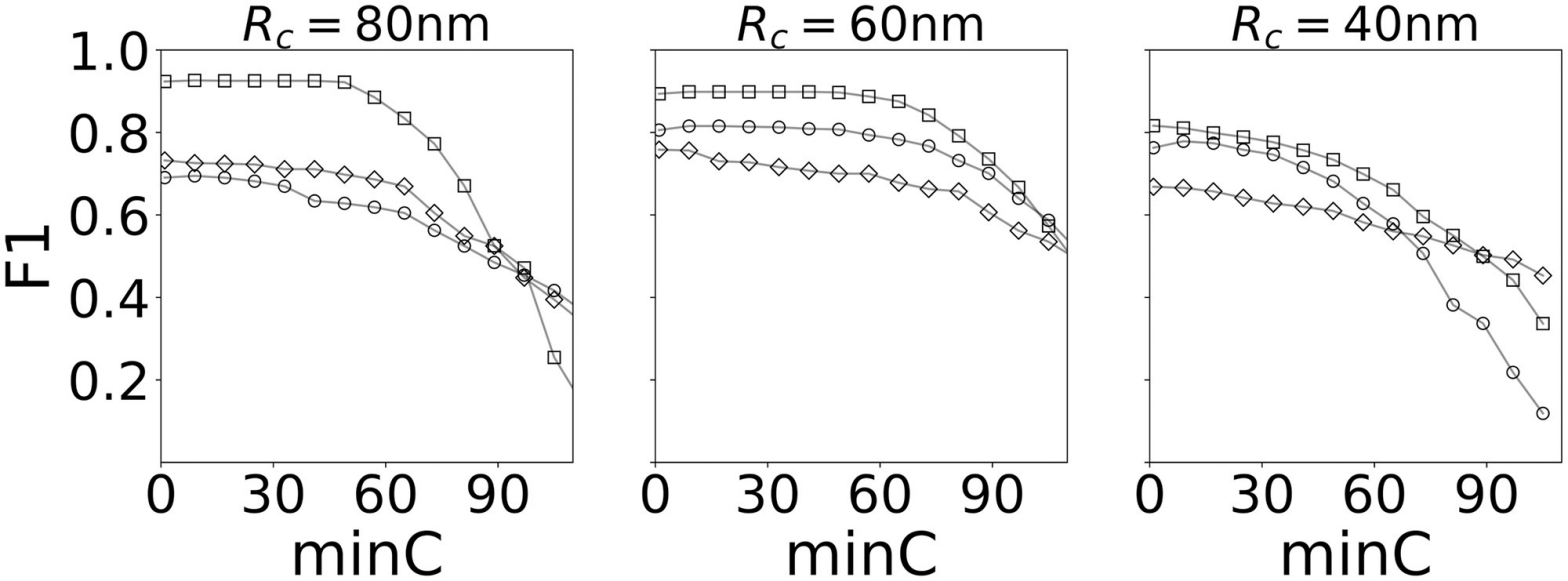
Clustering performance as indicated by the F1 score. (Corresponding to the analysis in Fig 3). The plots show that the algorithm is most accurate when identifying clusters within the plateau region (compare to Fig 3) near a grid spacing Δ*. Symbols the same as indicated in Fig 3.

We also find that the calculated F1 scores, displayed in Fig 4, are consistently the highest along the plateau at the optimized grid size (Δ* = 40 nm), and are significantly poorer within this same region for both the coarse (Δ = 90 nm) and fine (Δ = 20 nm) grids (see S3 Fig). Furthermore, the F1 scores remain relatively constant along the plateau.

Similar behaviour is observed for smaller clusters, with the results for 60 nm and 40 nm clusters shown here (Fig 3). We note that as the mean cluster size is decreased, the region of insensitivity of the algorithm to *minC* diminishes. For 40 nm clusters, and an appropriate choice of grid size, a plateau is still visible, if just, in a plot of the number of clusters vs. *minC*. We also note that, due to the reduced localization precision along the z-axis, the 40 nm clusters are significantly more elongated than the 80 nm clusters. This tends to shift the choice of ideal grid size to larger values as seen in the figure. Moreover, in both cases the F1 scores are highest, and relatively constant, along the plateau region of the Δ* curve (Fig 4).

#### DBSCAN at moderate noise levels

**Fig 5:**
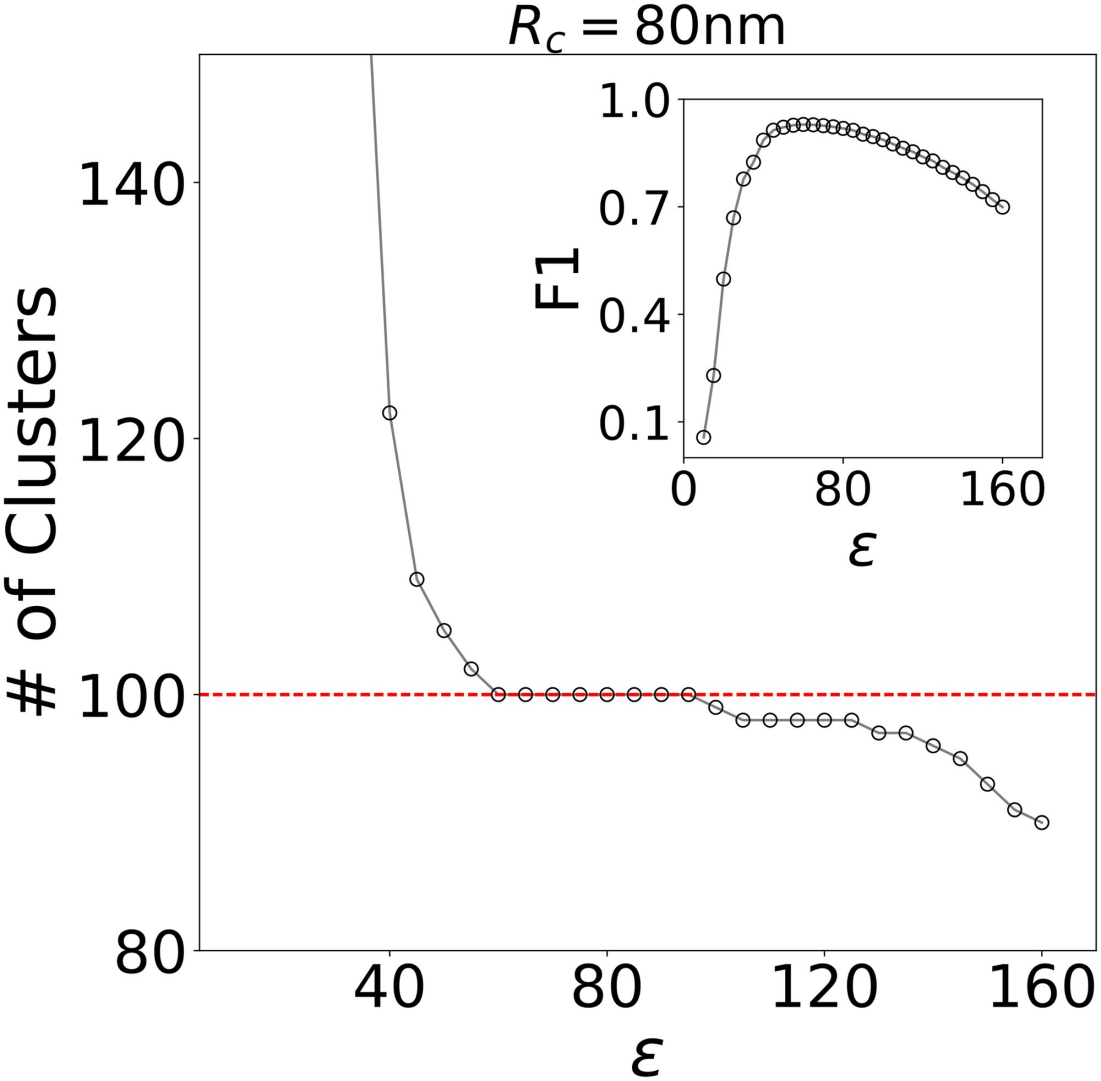
DBSCAN performance at *ζ* = 0.01 for target clusters of radius 80, 60 and 40 nm. Figures display the detected number of clusters vs. the length scale *ϵ* defining the local volume. The dashed line indicates the target number of clusters. Insert: F1 score vs. *ϵ*.

We next consider how DBSCAN performs on the same simulated data sets with an analogous optimization of input parameters. In Fig 5, for the case of 80 nm clusters, we plot the number of clusters detected by DBSCAN as a function of *ϵ*, which corresponds to *minC* in FOCAL3D (results for 60 nm and 40 nm clusters are provided in S4 Fig). At each point we have optimized the density threshold *minPts* to differentiate from random background noise, similar to the corresponding selection of *minL* in FOCAL3D.

For these simulations, DBSCAN also displays a clear plateau or region of insensitivity to the input parameter defining the local area (i.e. *ϵ*). However, the F1 scores appear to peak at the onset of the plateau, for lower values of *ϵ*, then steeply decline while still within the plateau region (see S5 Fig). Regardless, the plateau appearing in plots of cluster number vs. *ϵ* indicate a non-biased way to select the appropriate parameters for DBSCAN. As we will show, these regions of insensitivity may disappear in more complex datasets than those we have simulated, leaving the user without a guide for selecting the clustering parameters.

#### FOCAL3D performance under high noise conditions

**Fig 6:**
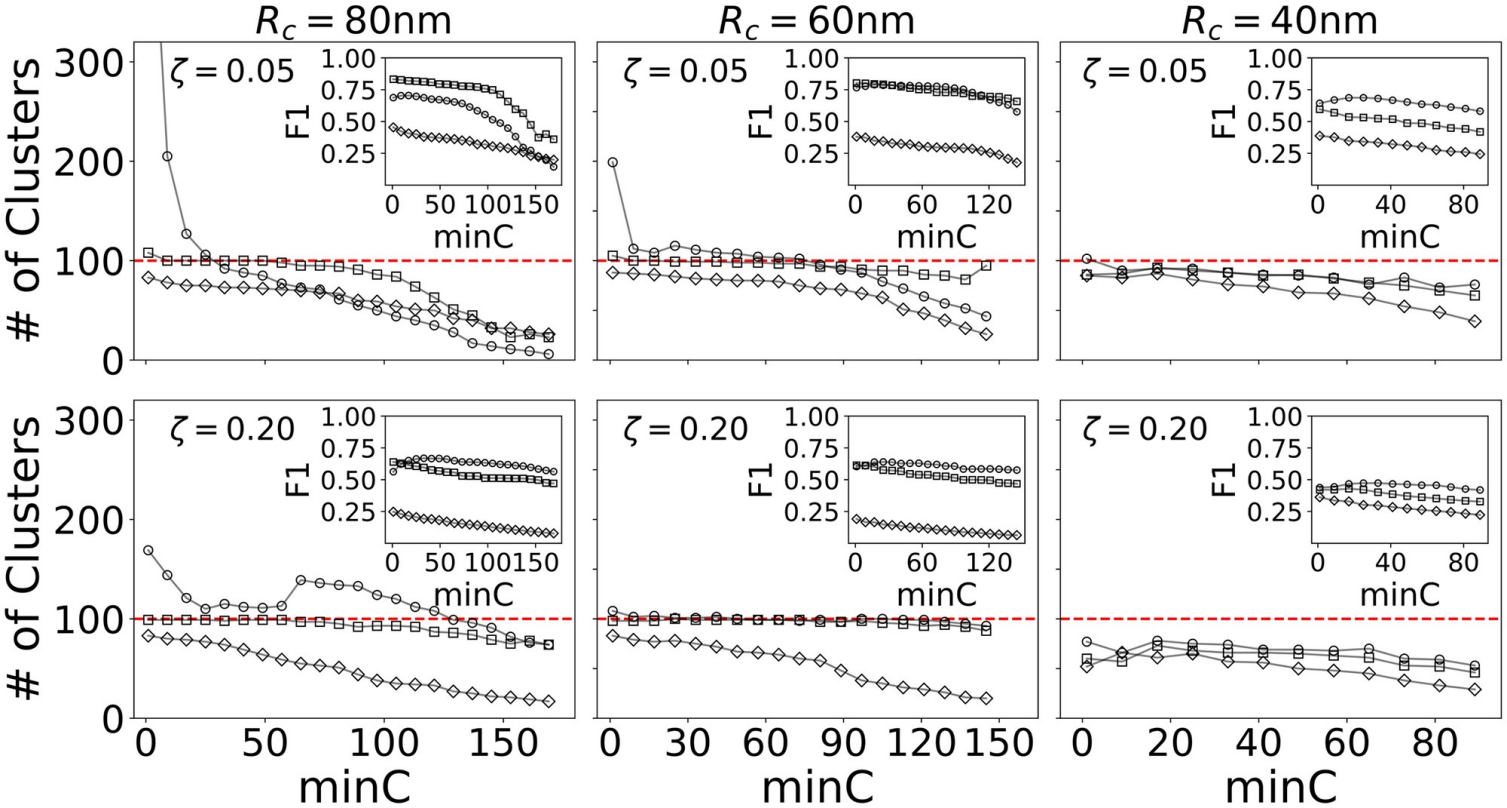
FOCAL3D performance at higher noise levels. *ζ* = 0.05 and *ζ* = 0.20 for 80 nm clusters: Δ = 20 nm (∘), Δ = 40 nm (▫), Δ = 90 nm (◊); 60 nm clusters: Δ = 20 nm (∘), Δ = 30 nm (▫), Δ = 80 nm (◊); 40 nm clusters: Δ = 20 nm (∘), Δ = 40 nm (▫), Δ = 60 nm (◊).

We now consider the performance of FOCAL3D under increasingly noisy conditions, both at *ξ* = 0.05 and *ξ* = 0.20. Our numerical results are displayed in Fig 6. For large 80 nm clusters and *ξ* = 0.05, signature curves appear in a plot of cluster number vs. *minC*. A steep descent is seen at overly fine grid sizes (Δ = 20 nm), giving way to a plateau near an optimized grid size (Δ* = 40 nm), which then drops below the target for coarser grids (Δ = 90 nm). We find that the region of insensitivity seen at Δ* = 40 nm holds even at very high noise levels (*ξ* = 0.20). FOCAL3D performs quite well for smaller clusters (*R*_*C*_ = 60 nm) under these noisy conditions and appears to actually show less sensitivity to the choice of target grid size. However, the algorithm begins to underestimate the number of target clusters for the smallest clusters we simulated (*R*_*C*_ = 40 nm), resulting in significantly poorer performance as the noise is increased from *ξ* = 0.05 to *ξ* = 0.20.

Again, at the target grid size Δ*, the F1 scores all display a relatively flat, maximum throughout the corresponding plateau region of *minC* (see S6 Fig). We note that at these higher noise levels the F1 scores lower, in part, because the background noise in the simulations is randomly distributed throughout the volume. For example, at *ξ* = 0.20, roughly 20% of the points contained within a cluster are considered background noise. Even if the algorithm were to perfectly identify the clusters, it would have no way to discern that these points are noise, resulting in an increased number of false positives.

#### Performance on mixed populations

To illustrate the performance of FOCAL3D at identifying heterogenous clustering, we analyzed simulated data sets with mixed populations of clusters of different mean radii and at moderate noise levels (*ζ* = 0.01). We considered three mixtures: 1) a 50/50 mixed population of mean radii *R*_*c*_ = 40 ± 8 nm and *R*_*c*_ = 80 ± 16 clusters (100 clusters in total), 2) a 50/50 mixed population of *R*_*c*_ = 60 ± 12 nm and *R*_*c*_ = 80 ± 16 clusters (100 clusters in total), and 3) a mixed population of *R*_*c*_ = 40 ± 8, *R*_*c*_ = 60 ± 12 nm, and *R*_*c*_ = 80 ± 16 nm clusters in equal proportion (40/40/40 for a total of 120 clusters). The average density of localizations in the clusters was the same as for the single population data sets at each cluster radii, and the centers of each cluster were separated by at least 250 nm. Despite the clear heterogeneity, the parameter scans continued to yield regions of insensitivity to *minC* at an optimal grid size Δ* for each of these mixed populations (see S7 Fig). Parameters chosen along these plateaus not only correctly detect the number of clusters but continue to display the highest F1 Scores (see S8 Fig) indicating the accuracy of FOCAL3D at identifying heterogeneous clustering. This is further confirmed by directly visualizing the cluster analysis (see S9 Fig). Any clustering errors tended to involve the sparse localizations at the edges of the clusters that resulted from the localization precision used to scatter the localizations within each cluster.

### Run-time scaling comparison

**Fig 7:**
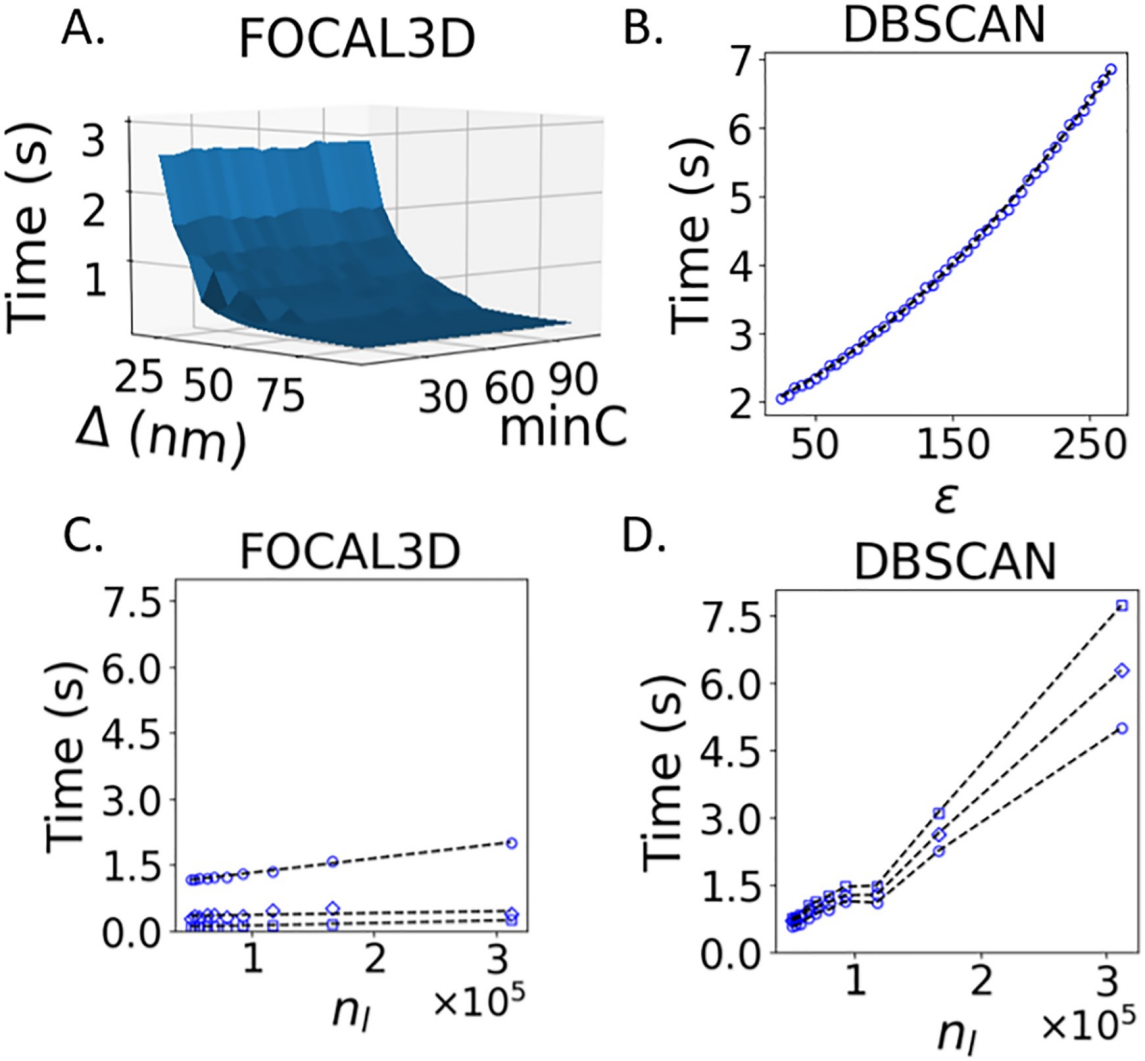
Run-time comparison between FOCAL3D and DBSCAN. Clustering of simulated data of 80 nm clusters ($\zeta =0.01,n_{l}^{max}=166274$ localizations). A) Run-time scaling of FOCAL3D for varying local volume *minC* and grid size Δ. B) Scaling of DBSCAN for varying local volume *ϵ*. C) Scaling of FOCAL3D for a varying number of localizations and at fixed *minC* = 15 for: Δ = 25 nm (∘), Δ = 45 nm (◊), and Δ = 65 nm (▫). D) Scaling of DBSCAN for a varying number of localizations at: *ϵ* = 40 nm (∘), *ϵ* = 70 nm (◊) and *ϵ* = 100 nm (▫). These values were chosen to roughly match the local volumes used in the FOCAL3D run-time analysis through the relation *minC* * Δ^3^ = (4*π*/3)*ϵ*^3^.

An important characteristic of any clustering algorithm is the run-time performance and scaling as a function of the size of the data set. Algorithms with a low run-time would expedite the analysis of multiple, large SMLM data sets, which are often needed to obtain the necessary statistics of a cluster phenotype. This is particularly relevant for three-dimensional SMLM data sets that typically have on the order of hundreds of thousands to millions of localizations per acquisition. In view of this, we directly compare the run time of FOCAL3D and DBSCAN as a function of their respective parameters and as a function of the number of localizations (*n*_*l*_) in the data set. We performed this analysis on simulated data of 80 nm clusters ($\zeta =0.01,n_{l}^{max}=166274$ localizations), and made use of a desktop computer housing an i5 quad-core single processor with 24 Gb of memory. Our results are summarized in Fig 7.

We first consider the run-time scaling of the two algorithms by varying the local volumes (i.e. *minC* and *ϵ* for FOCAL3D and DBSCAN, respectively). The run-time for FOCAL3D stays relatively constant when increasing *minC* (Fig 7A), compared to the rapid power law scaling displayed by DBSCAN (Fig 7B) for increasing *ϵ*. However, while varying the grid size Δ in FOCAL3D hardly affects this observed independence of the run-time scaling on *minC*, it does greatly slow down the algorithm for increasingly small grids.

A further comparison is provided by matching the local volumes employed by the two algorithms with the relation *minC* * Δ^3^ = (4*π*/3)*ϵ*^3^. FOCAL3D’s run-time scales linearly with the number of localizations in the data set, resulting in a time complexity of *O*(*n*_*l*_) (Fig 7C). Moreover, the rate at which the run-time increases with data set size is lower for larger grid sizes. This is in contrast with DBSCAN, whose run-time has been shown to scale as *O*(*n*_*l*_ log *n*_*l*_) at best [11]. For the Python implementation tested here (Scikit-learn), the run-time scaling appears even more complex and significantly worsens above a critical number of localizations (Fig 7D).

### Clustering of the nuclear pore complex

We now apply our clustering algorithm to 3-dimensional astigmatic dSTORM images of the nuclear pore complex (NPC) [21–23]. This will illustrate the validity of the parameter optimization as well as the ability of the algorithm to cluster complex biological data. The NPCs serve as clustering targets that are both heterogeneous, due to a reported labeling efficiency of only ∼ 55%, and complex in that they are of an extended, ring-like shape. The images were acquired in wild-type U-2 human osteosarcoma (OS) cells expressing Nup107-SNAP, which were fluorescently labeled with the organic dye Alexa-647 and induced to photoswitch by modifying the imaging buffer. Details on the cell cultures, labeling, fixation, and imaging can be found in [21].

**Fig 8:**
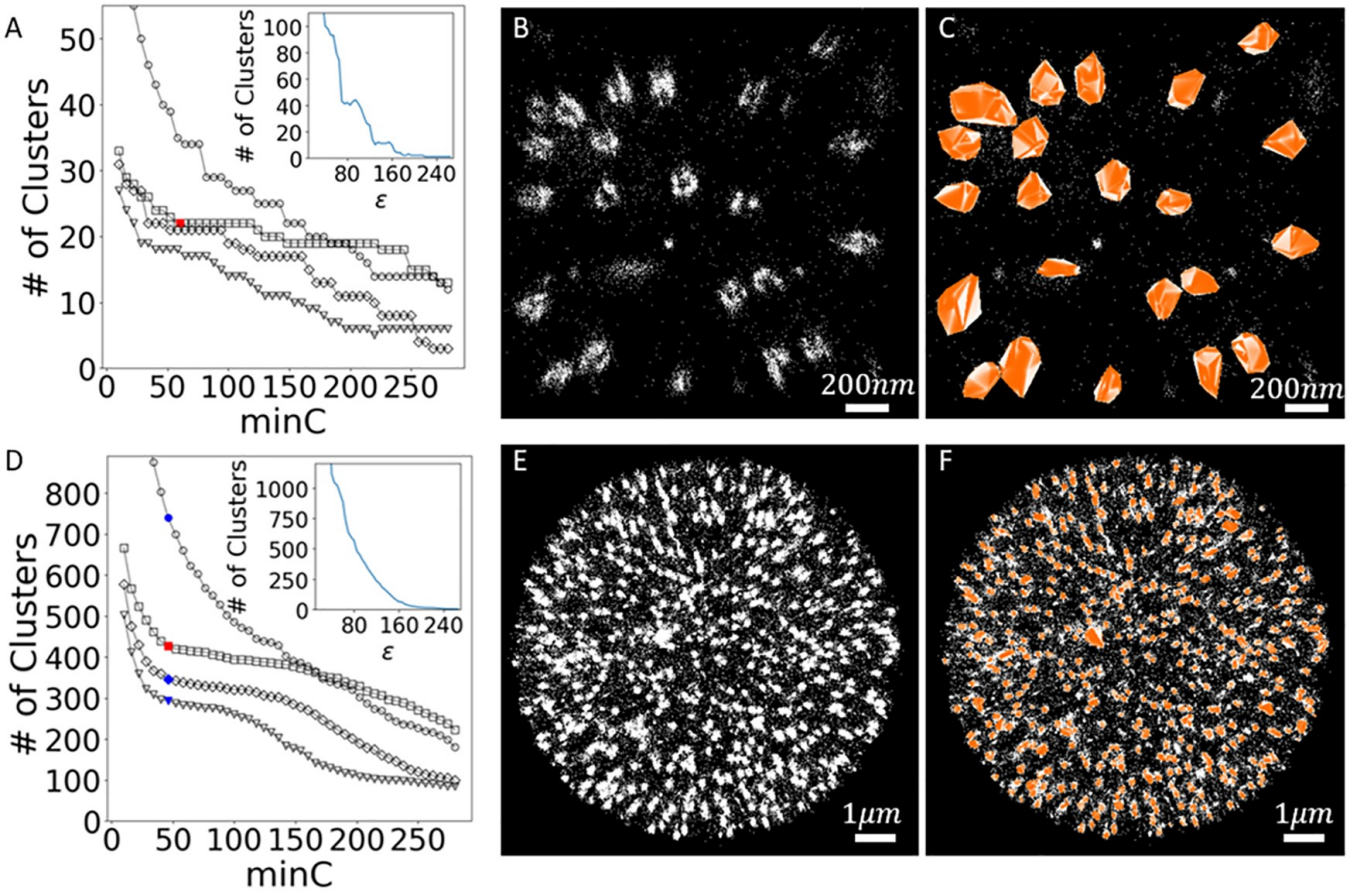
Cluster analysis of NPC dataset in U-2 OS cells. A) FOCAL3D analysis of the number of Clusters vs. *minC*, for the cropped dataset, for grid sizes of Δ = 15 nm (∘), 25 nm (▫), 35 nm (◊), 45 nm (∇). Insert: Corresponding DBSCAN analysis of the # of Clusters vs. *ϵ*. B) Localization data. C) FOCAL3D clustered dataset at Δ* = 25 nm and minC* = 60. D) FOCAL3D analysis of # of Clusters vs. *minC* for the full dataset. Symbols are the same as in the cropped dataset. Insert: Corresponding DBSCAN analysis of the # of Clusters vs. *ϵ*. E) Localization data. F) FOCAL3D clustering results at minC* = 46 and Δ* = 25.

We initially partition the dataset focusing on an approximately 2 *μ*m × 2 *μ*m region in the lateral plane centred on the nucleus. Fig 8A, 8B and 8C show the results of the clustering analysis with FOCAL3D. In Fig 8A, we scan through grid sizes ranging from 15-45 nm in steps of 10 nm. The smallest grid size (Δ = 15 nm) shows a steep decline in the number of clusters detected as a function of *minC*. This dependence significantly levels out at a grid size of Δ = 25 nm, with both the extent of the flat region and the estimated number of clusters decreasing for larger choices of the grid size. The discrete, step-like behaviour seen in the curves is simply an artifact of the small number of clusters that we are considering here (we, therefore, identify the plateau as the initial step after the inflection point of the curves). We identify Δ* = 25 and choose minC* = 60, which is just at the cusp of the plateau (red square). Fig 8B displays the localizations for this subset of the data, projected onto the x-y plane, in which we can manually identify NPCs by eye. A comparison with the clustering results (Fig 8C) nicely illustrates the performance of FOCAL3D. Note, the clustering is not strongly dependent upon the choice of grid size for a range of Δ*. For instance, we could have, alternatively, chosen Δ* = 35, which at minC* = 60 identifies roughly 1 less NPC and clusters the localizations in a similar fashion.

We also attempted to guide a similar analysis with DBSCAN by generating a plot of the number of detected clusters vs. *ϵ* (insert to Fig 8A). However, the results showed a steep functional dependence with no clear indication of how to select for *ϵ*. While a number of steps or plateaus appeared in this plot, the most extensive ones occurred far from the target number of clusters (around N = 10 and 40).

In Fig 8D, 8E and 8F we consider the full NPC dataset in which a manual enumeration of the NPCs would be difficult. Fig 8D shows an analysis of the detected number of clusters vs. *minC* over the same range of grid sizes (Δ = 15 − 45 nm) as analyzed for the smaller data subset. Once again, an overly fine grid size (Δ = 15 nm) shows a steep dependence on *minC*, with the curves levelling off at Δ = 25 nm, after which the plateau region shrinks and the number of clusters identified for a given *minC* drops. Again, we provide a localization image (Fig 8E), shown as a projection onto the x-y plane, that can be compared to the clustering results (Fig 8F). Here we have chosen Δ* = 25 and minC* = 46 (red square). A similar analysis for DBSCAN gives no indication of an appropriate way to select for *ϵ* (insert of Fig 8D).

For this larger dataset, we note that there is a certain level of ambiguity in the choice of Δ*, and the clustering that results is more sensitive to this choice. The effect is primarily due to the close proximity of many of the clusters, a facet that was not explored in our simulations where we explicitly prevented the clusters from overlapping. This can be quantified to some extent by the silhouette score *S*_*C*_ (see S1 Table). For example, in the simulations shown in Fig 3, optimal clustering resulted in a silhouette score within the range of *S*_*C*_ = 0.80 − 0.85. For the restricted NPC dataset shown in Fig 8A and 8B, *S*_*C*_ = 0.53 at Δ* = 25 nm. However, at the inflection points along the other curves in Fig 8D, indicated by the shaded blue symbols at minC* = 46, we find *S*_*C*_ = 0.48, 0.46, and 0.42 for Δ = 15 nm, 35 nm, and 45 nm, respectively. This indicates that within the image, on average, the predicted clusters are significantly more tightly packed.

FOCAL3D was not designed to distinguish overlapping clusters, so we would expect it to have difficulty with such a dataset. To better understand the behaviour of our algorithm, however, and to further guide us in our parameter selection, we considered the distribution of cluster radii detected at the cusp of each plateau in Fig 8D (indicated by the shaded red and blue points in the figure). Starting from the smallest grid size and increasing, the distribution in cluster radii tend to shift toward a peaked distribution, but then develop an increasingly long tail containing larger clusters (see S10 Fig). The latter effect most likely arises when the algorithm begins to merge the clusters, so the ideal clustering should occur just before this tail develops. This is supported by direct visualization of the clustering results (see S11 Fig). This is further justification that our choice of Δ* = 25 was appropriate. As a final check, the peak of the distribution in cluster sizes agrees with a Ripley’s K-function analysis (see S12 and S13 Figs, respectively).

## Discussion

As SMLM continues to push the resolution limit obtainable by light microscopy, efficient algorithms need to be developed that can cope with the concomitantly larger datasets that will be generated. In this manuscript, we have presented an extension of our 2-dimensional Fast Optimized Clustering Algorithm for Localization Microscopy (FOCAL) [14] to 3-dimensions. FOCAL3D is a density based algorithm, so directly identifies clusters in noisy SMLM images, and outperforms the classical density based algorithm DBSCAN both in terms of performance (scaling like *n*_*l*_ compared to *n*_*l*_ log *n*_*l*_) and ability to identify complex, heterogeneous clusters. Moreover, unlike DBSCAN, the algorithmic parameters that determine FOCAL3D’s performance can be systematically tuned such that, within a constrained range of parameters, the clustering is only weakly dependent upon the exact choice of these parameters.

While the speed gains of working on a grid can be considerable, for small clusters that necessitate an excessively fine grid, the computational cost of FOCAL3D can outweigh that of DBSCAN. As we saw in our simulations, the region of parametric insensitivity is also reduced making it harder to optimize the cluster detection. However, the localization precision of SMLM, which is typically on the order of 10s of nanometers, effectively sets a lower limit to the grid size [14]. This is because all photoswitchable or photoactivatable fluorophores tend to blink, with the same dye giving rise to multiple localizations. In quantitative SMLM, which attempts to quantify the abundance of nucleic acids or proteins from SMLM data, blinking gives rise to an overcounting problem [24–28]. In a clustering analysis, unclustered molecules with a single fluorophore label may appear as small clusters, of a size determined by the localization precision, due to blinking. For most cluster detection problems, the optimal grid size and the cluster threshold *minC* will exclude blinking artefacts from the cluster analysis.

Finally, FOCAL3D may have issues analyzing dense SMLM image reconstructions, particularly when the clusters begin to overlap. Future extensions to FOCAL3D may alleviate these issues, such as by incorporating a segmentation algorithm to differentiate anomalously large, dense clusters of localizations.

FOCAL3D is designed to automate and rapidly perform an analysis of large 3-dimensional SMLM data sets. Likewise, the resulting clustering may be used as a way to filter out noise in SMLM image reconstructions, retaining only the features of interest. FOCAL3D should serve as a useful addition to the set of quantitative techniques now available for super-resolved microscopy, providing a foundation for further analysis of intracellular organization, protein assemblages, and spatial patterning.

## Supporting information

S1 Text Supporting text. Practical aspects of the clustering optimization and simulation details.
(PDF)

S1 Table Supporting table. Silhouette Scores from the clustering of the NPC dataset.
(PDF)

S1 Fig Simulated ground truth cluster sizes. Simulated ground truth cluster radii obtained by convex hull for A) 80 ± 16 nm clusters, B) 60 ± 12 nm clusters, C) 40 ± 8nm clusters. Solid black line is a Gaussian fit to the peak of the distribution.
(TIF)

S2 Fig Insensitivity to grid selection of clustering. There is some flexibility on the choice of optimal grid size as illustrated in these graphs, which correspond to the results from Fig 3 of the main text. FOCAL3D performance for simulations at a noise level of *ζ* = 0.01. For *R*_*c*_ = 80 nm: Δ = 35 nm (∇), Δ = 40 nm (▫), Δ = 45 nm (Δ). For *R*_*c*_ = 60 nm: Δ = 25 nm (∇), Δ = 30 nm (▫), Δ = 35 nm (Δ). For *R*_*c*_ = 40 nm: Δ = 30 nm (∇), Δ = 40 nm (▫), Δ = 50 nm (Δ).
(TIF)

S3 Fig FOCAL3D Precision and Recall at moderate noise. Precision and Recall curves used to calculate the F1 Scores in Fig 4 of the main text (*ζ* = 0.01).
(TIF)

S4 Fig DBSCAN performance with smaller clusters. DBSCAN performance at detecting 60 nm and 40 nm clusters at moderate noise (*ζ* = 0.01 simulations).
(TIF)

S5 Fig DBSCAN Precision and Recall at moderate noise. Precision and Recall curves used to calculate the F1 Scores in the inset of Fig 5 of the main text (*ζ* = 0.01 simulations).
(TIF)

S6 Fig FOCAL3D Precision and Recall at high noise. Precision and Recall curves used to calculate the F1 Scores for *ζ* = 0.05 and *ζ* = 0.20. Shown are the results for cluster sizes of 80 nm, 60 nm and 40 nm in Fig 6 of the main text.
(TIF)

S7 Fig Parameter selection for mixture of different cluster sizes. FOCAL3D performance for simulated populations of mixed cluster sizes at a noise level of *ζ* = 0.01. At each radius, the number of clusters as a function of *minC* is displayed for a range of grid sizes. (Mixture of *R*_*c*_ = 80 nm and *R*_*c*_ = 40 nm: Δ = 20 nm (∘), 40 nm (▫), 80 nm (◊); mixture of *R*_*c*_ = 80 nm and *R*_*c*_ = 60 nm: Δ = 20 nm (∘), 45 nm (▫), 80 nm (◊); mixture of *R*_*c*_ = 80 nm, *R*_*c*_ = 60 nm, and *R*_*c*_ = 40 nm: Δ = 20 nm (∘), 35 nm (▫), 80 nm (◊)). The red, dashed line indicates the actual number of simulated clusters: 100 and 120.
(TIF)

S8 Fig F1 Scores for mixture of different cluster sizes. F1 Scores for mixture of *R*_*c*_ = 80 nm and *R*_*c*_ = 40 nm: Δ = 20 nm (∘), 40 nm (▫), 80 nm (◊); mixture of *R*_*c*_ = 80 nm and *R*_*c*_ = 60 nm: Δ = 20 nm (∘), 45 nm (▫), 80 nm (◊); mixture of *R*_*c*_ = 80 nm, *R*_*c*_ = 60 nm, and *R*_*c*_ = 40 nm: Δ = 20 nm (∘), 35 nm (▫), 80 nm (◊). For simulations at *ζ* = 0.01.
(TIF)

S9 Fig Visualization of cluster analysis for mixture of different simulated cluster sizes. Visualization of the mixed population data set (40 clusters of 40 nm, 60 nm and 80 nm cluster sizes each, for a total of 120 clusters) at moderate noise levels (*ζ* = 0.01). A. Raw data with noise localizations included. B. Raw cluster data with noise localizations removed (for visualization purposes). C. FOCAL3D clustering results at *minC* = 13, Δ = 35, as identified from S7 Fig, with noise localizations removed for visualization purposes. D. FOCAL3D results showing only clustered localizations (noise localizations removed for visualization purposes). A few of the localizations at the edge of each cluster are missed, but FOCAL3D precisely and accurately identifies 120 of the clusters (out of 120).
(TIF)

S10 Fig Cluster radius vs. grid spacing for NPC dataset. Effective cluster radii for NPC dataset at different grid sizes (evaluated at *minC* = 46 in Fig 8). For increasing grid size, the distribution in cluster radii first shifts toward a peaked distribution. Then for larger grids, this peaked distribution gradually diminishes while increasingly extending a long tail (indicating large clusters). This is due to separate, but neighbouring, NPCs being grouped into the same cluster.
(TIF)

S11 Fig NPC cluster analysis at non-optimal parameters. FOCAL3D clustering results at *minC* = 46 and grid sizes Δ = 15 nm, 35 nm, and 45 nm. The optimal grid size, shown in the main text, was selected to be Δ = 25. For too small of a grid size (Δ = 15 nm), FOCAL3D misses many clusters. For larger grid sizes (Δ = 35 nm and 45 nm), FOCAL3D joins neighbouring distinct clusters together, indicating sub-optimal performance for this data set.
(TIF)

S12 Fig Cluster radius vs. minC for the NPC dataset. Effective cluster radius, determined by convex hull, for NPC dataset at different values of minC (Δ* = 25 nm). A similar behaviour is observed as in the simulations. For small *minC*, several false small clusters are identified. About some optimal value, we find a single peaked distribution. And for too large a choice of *minC*, reasonable, smaller clusters begin to get cut by the size threshold. The average cluster size for a *minC* of 46 is 109 ± 39.
(TIF)

S13 Fig Effective cluster radius from Ripley’s H-function for the NPC dataset. Ripley’s H-function (left) and its derivative (right) for the NPC data set. The cluster size is estimated to be about 132 nm.
(TIF)
