# Supplementary material for: FOCAL3D: A 3-dimensional clustering package for single-molecule localization microscopy
Source: PLoS Comput Biol. 2020 Dec 8;16(12):e1008479. doi: 10.1371/journal.pcbi.1008479 (PMC7748281; doi:10.1371/journal.pcbi.1008479)
Supplement: S1 Text — Practical aspects of the clustering optimization and simulation details. (PDF) [file pcbi.1008479.s001.pdf]

Supporting Information S1 Text

"FOCAL3D: A 3-dimensional clustering package for single-molecule localization microscopy"

Daniel F. Nino[1,2], Daniel Djayakarsana[2], and Joshua N. Milstein[1,2]

[1]Department of Physics, University of Toronto
[2]Department of Chemical and Physical Sciences, University of Toronto Mississauga

1 Clustering Optimization

1.1 Flowchart of FOCAL3D optimization

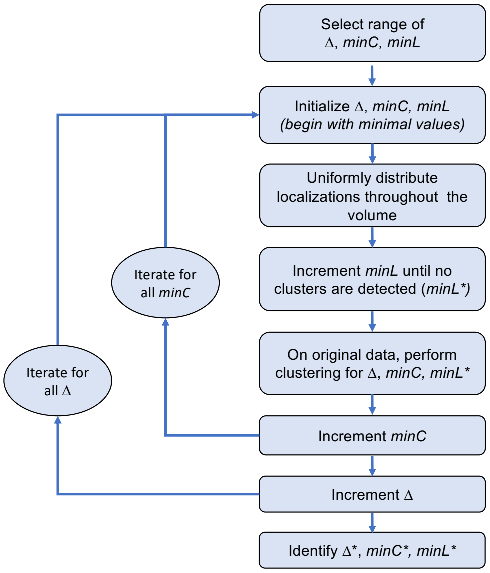

Fig 1: A flowchart illustrating the discussion in Section 2.2 on optimizing the parameters in FOCAL3D.

1.2 Lower bound of $minL$ in FOCAL3D

To save computation time in optimizing $minL$, we can narrow the search range by estimating a lower bound. We find an optimal $minL$ by randomly scattering all N_{total} localizations within the image volume V, so that the density of localizations is N_{total}/V. For a given grid size Δ, the volume occupied by a voxel is $\delta V = \Delta^3$ and the average number of localizations per voxel is $N_{total}\frac{\delta V}{V}$. Since the image is initially enhanced by locally summing all neighbouring voxels, we should multiply by the voxel connectivity ($3^3-1 = 26$ in 3D or $3^2 - 1 = 8$ in 2D). So, for the 3D case, we find a lower bound of:

$$minL > 26N_{total}\frac{\Delta^3}{V}. \tag{1}$$

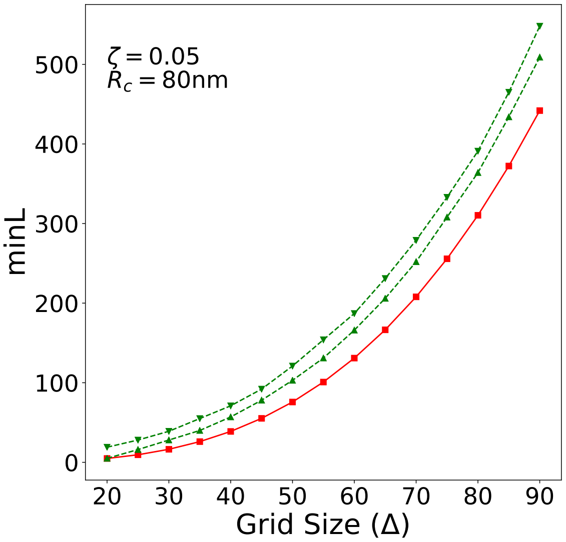

Fig 2: Estimating the lower bound of $minL$ in FOCAL3D. $\zeta = 0.05$ and a cluster size of 80 ± 16 nm within a 5 μm$\times$5 μm$\times$5 μm simulation volume. Estimated lower bound ($\square$), $minL^*$ at $minC = 5$ ($\triangledown$):, $minL^*$ at $minC = 141$ ($\triangle$)

1.3 Lower bound of $minPts$ in DBSCAN

We can also set a lower bound for $minPts$ in DBSCAN. Similar to the case for $minL$, we can estimate the average number of randomly scattered localizations we expect in a sphere of radius ϵ:

$$minPts > N_{total}\frac{4\pi\epsilon^3}{3V}. \tag{2}$$

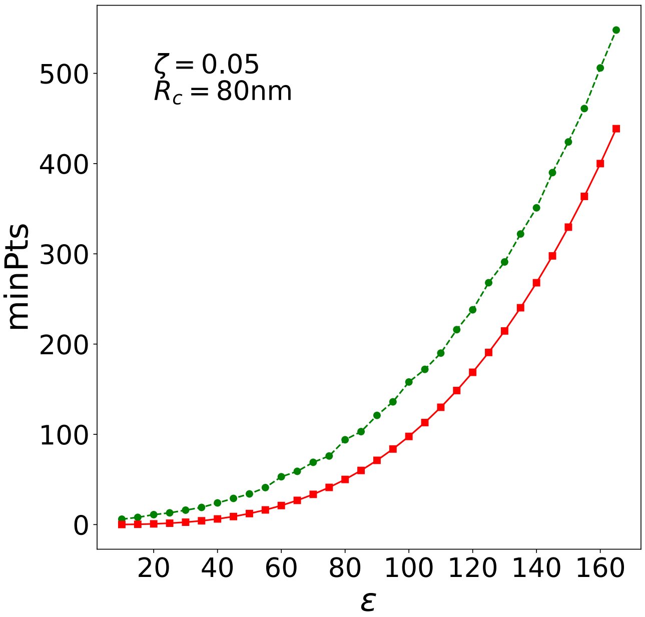

Fig 3: Estimating the lower bound of $minPts$ in DBSCAN. $\zeta = 0.2$ and a cluster size of 80 ± 16 nm within a 5 μm×5 μm×5 μm simulation volume. Estimated lower bound (□), $minPts^*$ (●).

2 Simulation Details

2.1 Distribution of the Total Number of Localizations per Fluorophore

The total number of localizations we obtain from a single, blinking fluorophore may be written as

$$n = n_1 + n_2 + \ldots n_{B+1}, \tag{3}$$

where $n_1, n_2, \ldots n_{B+1}$ are the number of localizations observed each time the fluorophore blinks up to a total of B blinks. Here we define a blink as a reactivation of the emissive state of the fluorophore.

Many fluorophores used in single-molecule localization microscopy are observed to have an ON time that follows an exponential distribution

$$p_{ON}(t) = \frac{1}{\tau_{ON}} e^{-t/\tau_{ON}}, \tag{4}$$

where τ_{ON} is the characteristic ON time for that fluorophore. If we work in units of the camera exposure time τ_{exp}, and identify $n \approx t/\tau_{exp}$ as the number of localizations that will be detected during a single emission period due to temporal binning by the camera, we can rewrite Eq. 4 as:

$$p_{ON}(n) \approx \frac{1}{\tilde{\tau}_{ON}} e^{-n/\tilde{\tau}_{ON}}, \tag{5}$$

where $\tilde{\tau}_{ON} = \tau_{ON}/\tau_{exp}$.

Since the sum of $B+1$ independent, identical exponential variables is an Erlang or Gamma Distribution, from Eq. 5, we can now derive the probability distribution for the total number of localizations n expected from a fluorophore that blinks B times:

$$f(n|B) = \frac{1}{\Gamma(B+1)\tilde{\tau}_{ON}^{B+1}} n^B e^{-n/\tilde{\tau}_{ON}}, \tag{6}$$

where $\Gamma(x)$ is the Gamma function (not to be confused with the Gamma distribution). However, the number of blinks B is also a statistical variable. Here we assume that B is properly modeled by a geometric distribution

$$p(B) = (1 - e^{-1/\lambda})e^{-B/\lambda}, \tag{7}$$

where λ is the characteristic number of blinks. This dependence has, likewise, been experimentally verified for a number of photoactivatable/photoswitchable fluorophores. With this in hand, we weigh the probability distribution $f(n|B)$, Eq. 6, by the probability of observing B blinks, Eq. 7, to obtain the distribution of the total number of localizations per fluorophore $p(n)$:

$$p(n) = \sum_{B=0}^{\infty} f(n|B)p(B) = (1 - e^{-1/\lambda})\frac{e^{-n/\tilde{\tau}_{ON}}}{\tilde{\tau}_{ON}} \sum_{B=0}^{\infty} \left(\frac{ne^{-1/\lambda}}{\tilde{\tau}_{ON}}\right)^B \frac{1}{B!}. \tag{8}$$

The sum is just the Taylor series expansion of e^x where $x = \frac{ne^{-1/\lambda}}{\tilde{\tau}_{ON}}$ in this case. Thus, the distribution of the total number of localizations per fluorophore is

$$p(n) = \frac{(1 - e^{-1/\lambda})}{\tilde{\tau}_{ON}} e^{-n(1-e^{-1/\lambda})/\tilde{\tau}_{ON}}, \tag{9}$$

which is an exponential distribution with a characteristic number of localizations per fluorophore of $\bar{n} = \frac{\tilde{\tau}_{ON}}{1-e^{-1/\lambda}}$. Since localizations are a discrete quantity, we can discretize the exponential distribution into a geometric distribution as

$$p(n) \sim \left(1 - e^{-(1-e^{-1/\lambda})/\tilde{\tau}_{ON}}\right) e^{-n(1-e^{-1/\lambda})/\tilde{\tau}_{ON}}. \tag{10}$$

2.2 Distribution of Localization Precision

We can model the distribution of localization precisions by taking into account the distribution of the total number of photons per blink:

$$p(n_p) = \frac{1}{\bar{n}_p} e^{-n_p/\bar{n}_p}. \tag{11}$$

An approximate lower bound for the localization precision is given by $\sigma_n \approx \sigma_{PSF}/\sqrt{n_p}$. From this we can find a model distribution of the localization precision:

$$\begin{aligned} p(\sigma_n) &= \int p(n'_p)\delta\left(\sigma_n - \frac{\sigma_{PSF}}{\sqrt{n'_p}}\right) dn'_p \\ &= \int \frac{1}{\bar{n}_p} e^{-n'_p/\bar{n}_p} \delta\left(\sigma_n - \frac{\sigma_{PSF}}{\sqrt{n'_p}}\right) dn'_p, \end{aligned} \tag{12}$$

where $\delta(x)$ is the Dirac delta function. With the substitution $u = \sigma_{PSF}/\sqrt{n'_p}$, this gives:

$$
\begin{aligned}
p(\sigma_n) &= \frac{2\sigma_{PSF}^2}{\bar{n}_p} \int \frac{1}{u^3} e^{-\frac{\sigma_{PSF}^2}{u^2 \bar{n}_p}} \delta(\sigma_n - u) du \\
&= \frac{2\sigma_{PSF}^2}{\bar{n}_p} \frac{1}{\sigma_n^3} e^{-\sigma_{PSF}^2/(\bar{n}_p \sigma_n^2)}.
\end{aligned}
\tag{13}
$$

.

The mode of this distribution can be calculated from the maximum likelihood estimate as:

$$
\delta = \sqrt{\frac{2}{3\bar{n}_p}} \sigma_{PSF}. \tag{14}
$$

The mean of this distribution is:

$$
< \sigma_n >= \sqrt{\frac{\pi}{\bar{n}_p}} \sigma_{PSF}. \tag{15}
$$

These relations are useful in scaling the axial localization precision with respect to the lateral precision. For example, by taking double the PSF width in the axial direction as in the lateral dimensions, we effectively shift the peak and mean of the distribution by a factor of 2.

To use this result in our simulations, we employ inverse transform sampling. The cumulative distribution function (CDF) of the localization precision is:

$$
F(\sigma_n) = \int_0^{\sigma_n} p(\sigma'_n) d\sigma'_n = e^{-\sigma_{PSF}^2/(\bar{n}\sigma_n^2)}, \tag{16}
$$

and the inverse CDF is:

$$
\sigma_n = F^{-1}(Y) = \sqrt{\frac{\sigma_{PSF}^2}{\bar{n}} \frac{1}{\ln(1/Y)}}, \tag{17}
$$

so if $Y \sim U(0,1)$, then $\sigma_n \sim p(\sigma_n)$.

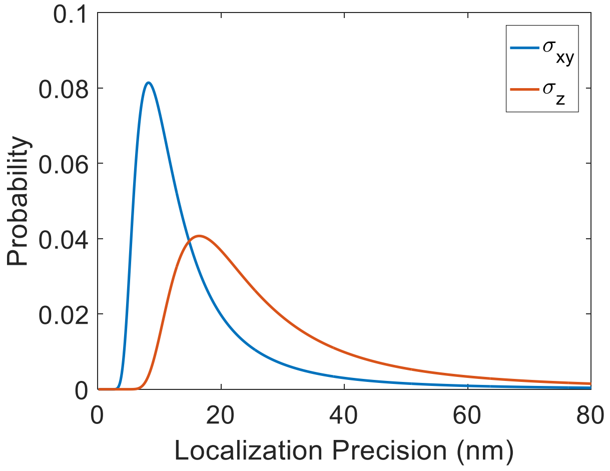

Fig 4: Theoretical localization precision distributions with $\bar{n}_p = 750$, and a point-spread function width of $\sigma_{PSF,xy} = 390$ nm in the lateral direction and $\sigma_{PSF,z} = 780$ nm in the axial direction. This results in modes of $\delta_{x,y} = 10$ nm in the lateral direction, and $\delta_z = 20$ nm in the axial direction.
