# Supplementary material for: FOCAL3D: A 3-dimensional clustering package for single-molecule localization microscopy
Source: PLoS Comput Biol. 2020 Dec 8;16(12):e1008479. doi: 10.1371/journal.pcbi.1008479 (PMC7748281; doi:10.1371/journal.pcbi.1008479)
Supplement: S1 Table — Silhouette Scores from the clustering of the NPC dataset. (PDF) [file pcbi.1008479.s002.pdf]

**Silhouette Score NPC**									
15	20	25	30	35	40	45	50	55	60
0.40	0.45	0.48	0.48	0.47	0.44	0.42	0.40	0.36	0.31
