# Supplementary figures and images for: FOCAL3D: A 3-dimensional clustering package for single-molecule localization microscopy
Source: PLoS Comput Biol. 2020 Dec 8;16(12):e1008479. doi: 10.1371/journal.pcbi.1008479 (PMC7748281; doi:10.1371/journal.pcbi.1008479)

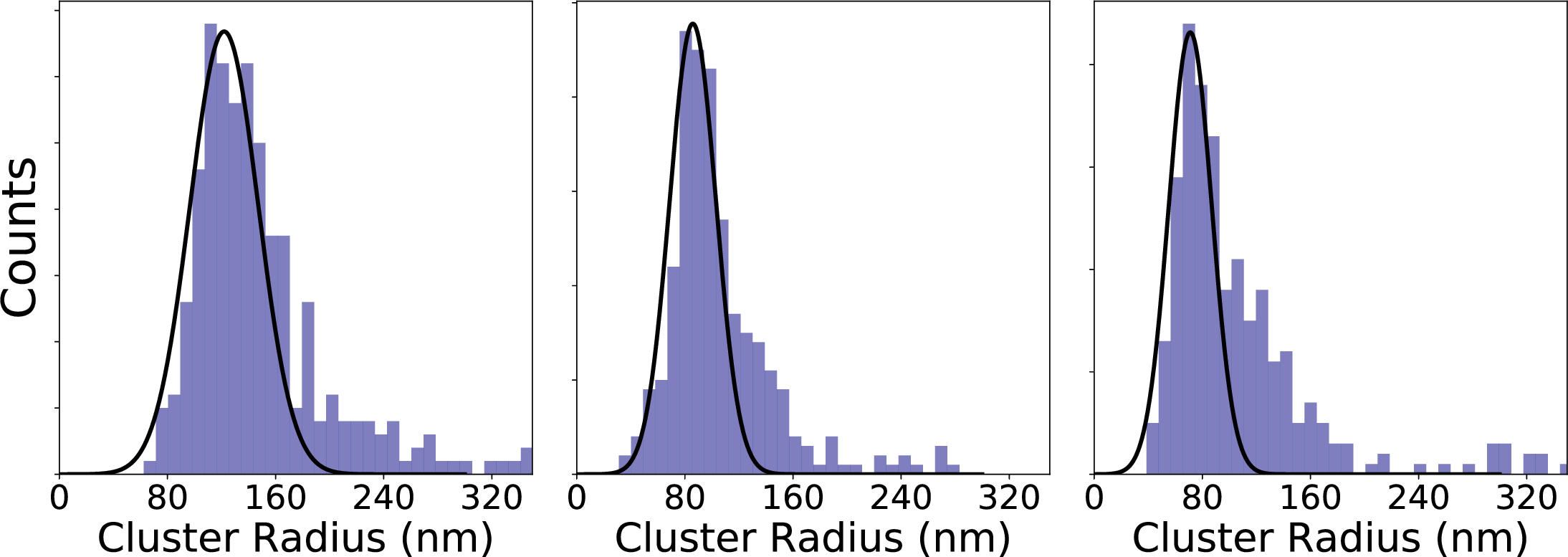

Supplement: S1 Fig — Simulated ground truth cluster radii obtained by convex hull for A) 80 ± 16 nm clusters, B) 60 ± 12 nm clusters, C) 40 ± 8nm clusters. Solid black line is a Gaussian fit to the peak of the distribution. (TIF) [file pcbi.1008479.s003.tif]

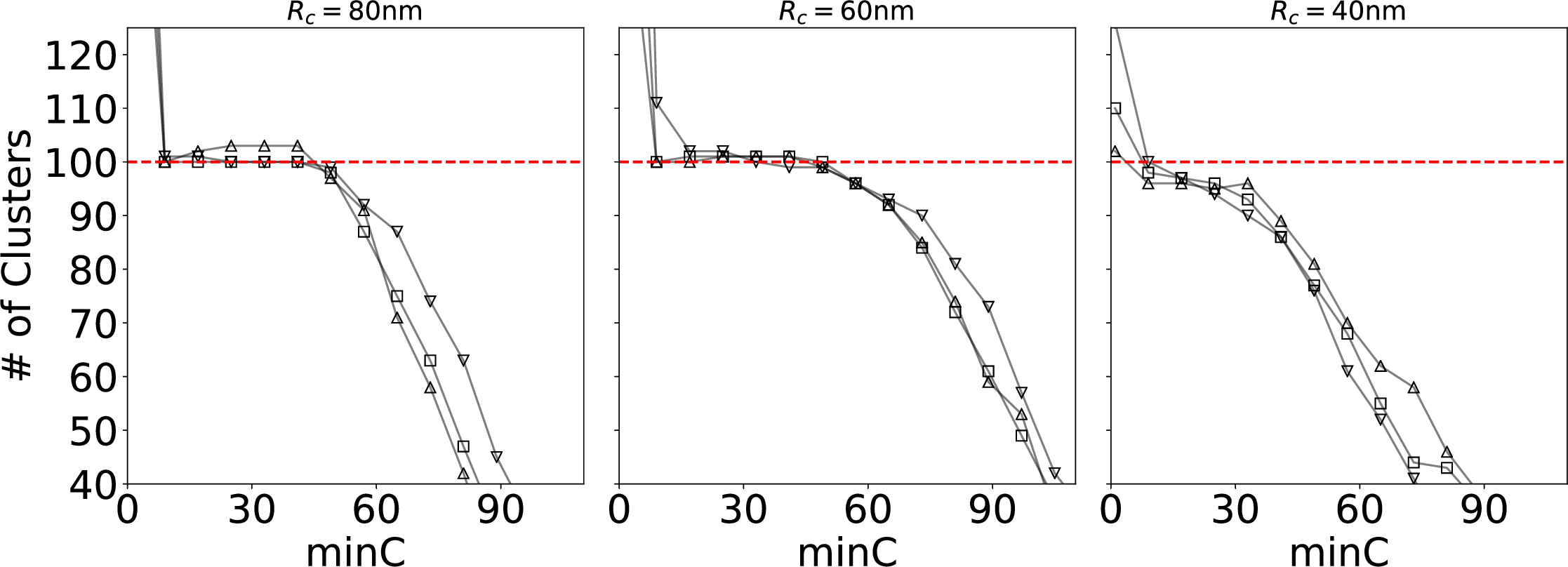

Supplement: S2 Fig — There is some flexibility on the choice of optimal grid size as illustrated in these graphs, which correspond to the results from Fig 3 of the main text. FOCAL3D performance for simulations at a noise level of ζ = 0.01. For Rc = 80 nm: Δ = 35 nm (∇), Δ = 40 nm (▫), Δ = 45 nm (Δ). For Rc = 60 nm: Δ = 25 nm (∇), Δ = 30 nm (▫), Δ = 35 nm (Δ). For Rc = 40 nm: Δ = 30 nm (∇), Δ = 40 nm (▫), Δ = 50 nm (Δ). (TIF) [file pcbi.1008479.s004.tif]

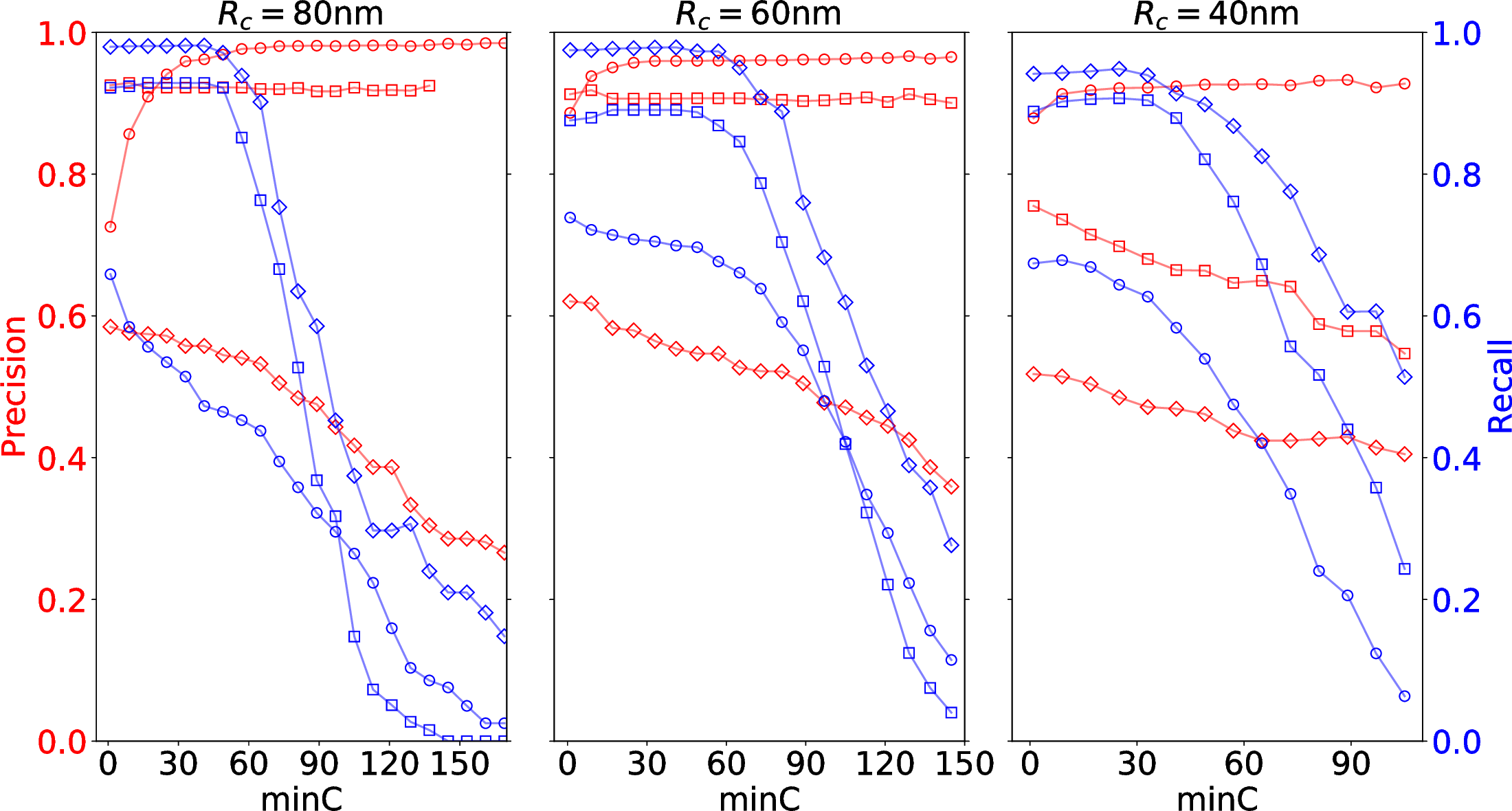

Supplement: S3 Fig — Precision and Recall curves used to calculate the F1 Scores in Fig 4 of the main text (ζ = 0.01). (TIF) [file pcbi.1008479.s005.tif]

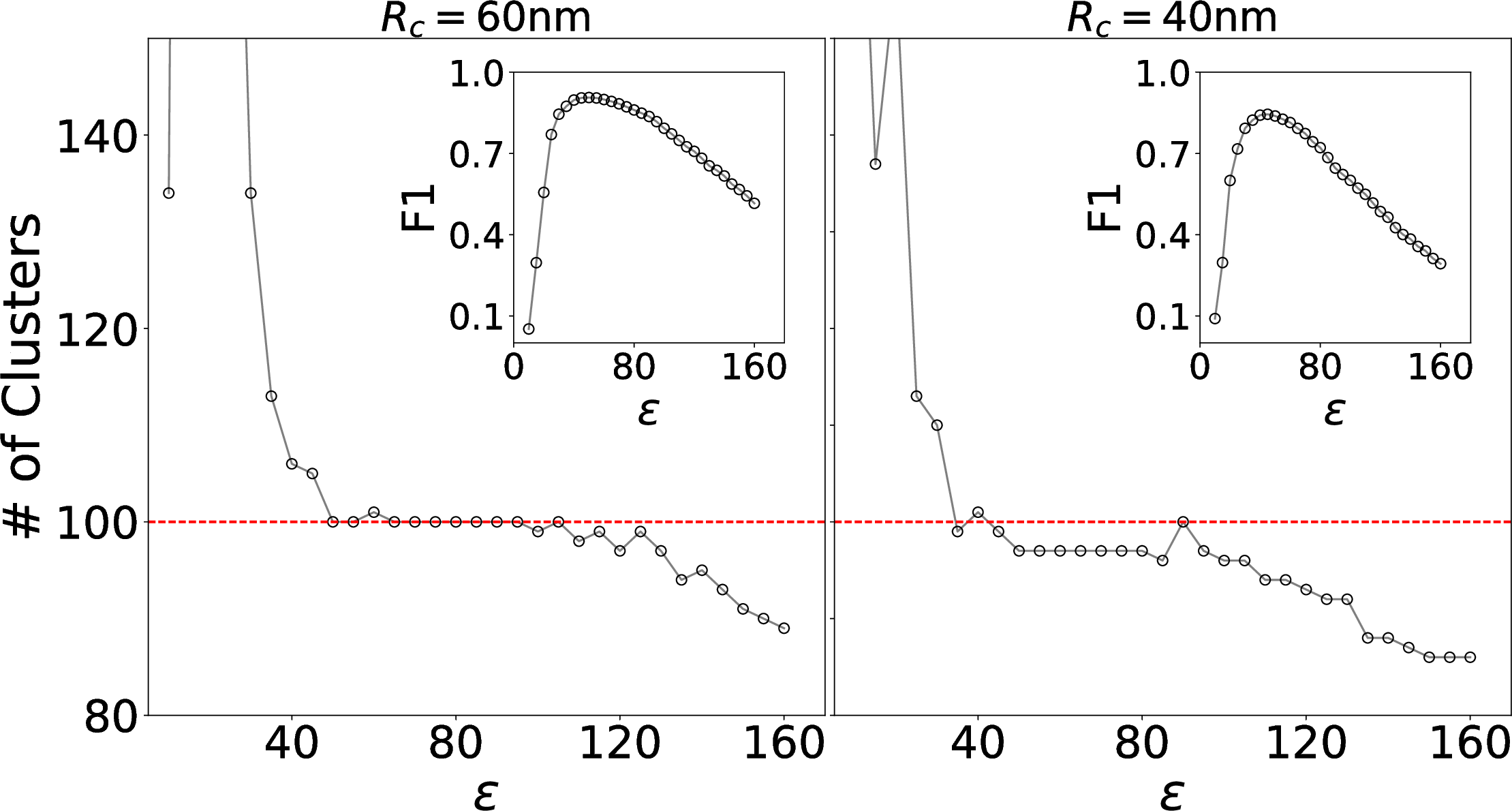

Supplement: S4 Fig — DBSCAN performance at detecting 60 nm and 40 nm clusters at moderate noise (ζ = 0.01 simulations). (TIF) [file pcbi.1008479.s006.tif]

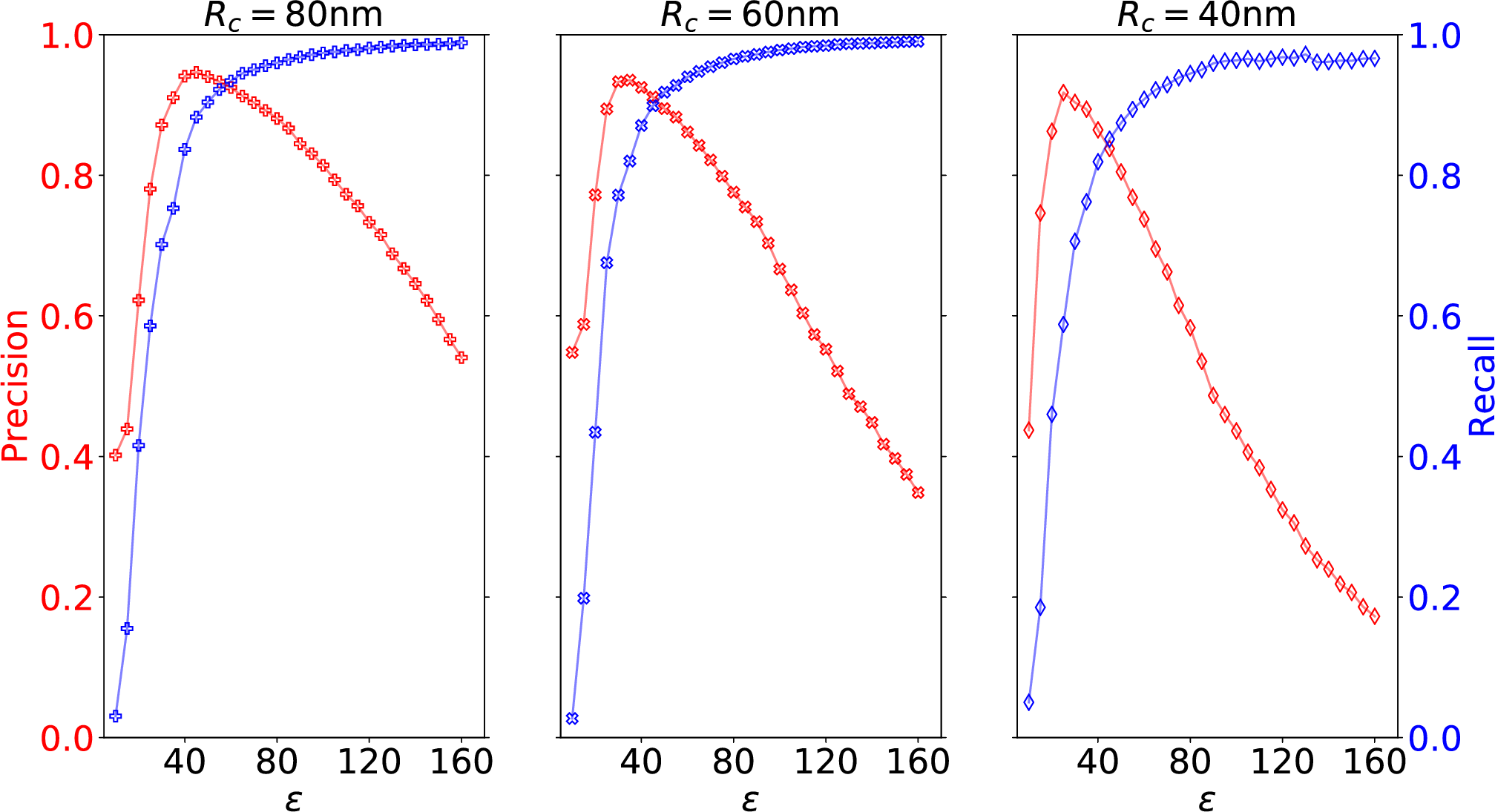

Supplement: S5 Fig — Precision and Recall curves used to calculate the F1 Scores in the inset of Fig 5 of the main text (ζ = 0.01 simulations). (TIF) [file pcbi.1008479.s007.tif]

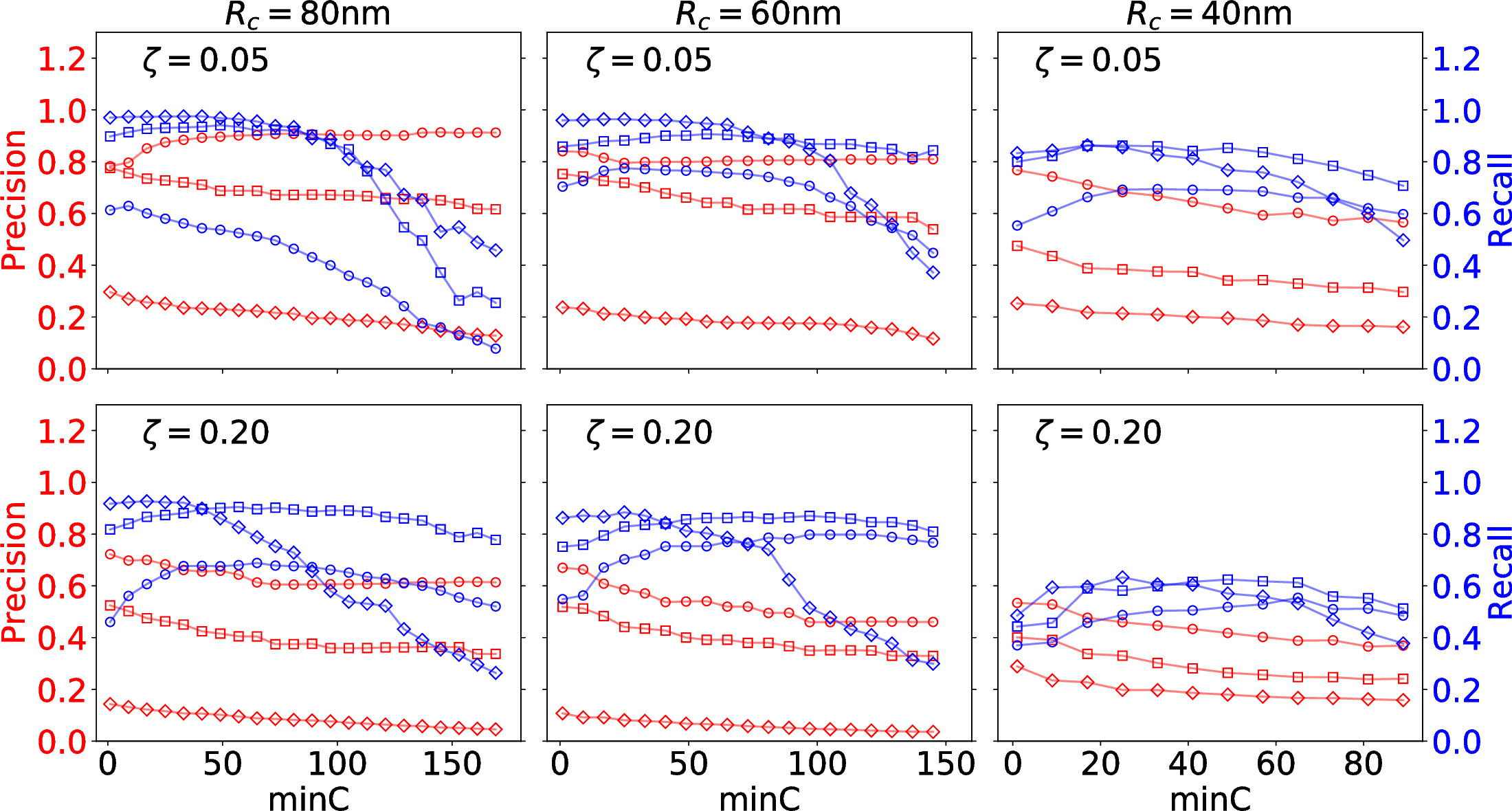

Supplement: S6 Fig — Precision and Recall curves used to calculate the F1 Scores for ζ = 0.05 and ζ = 0.20. Shown are the results for cluster sizes of 80 nm, 60 nm and 40 nm in Fig 6 of the main text. (TIF) [file pcbi.1008479.s008.tif]

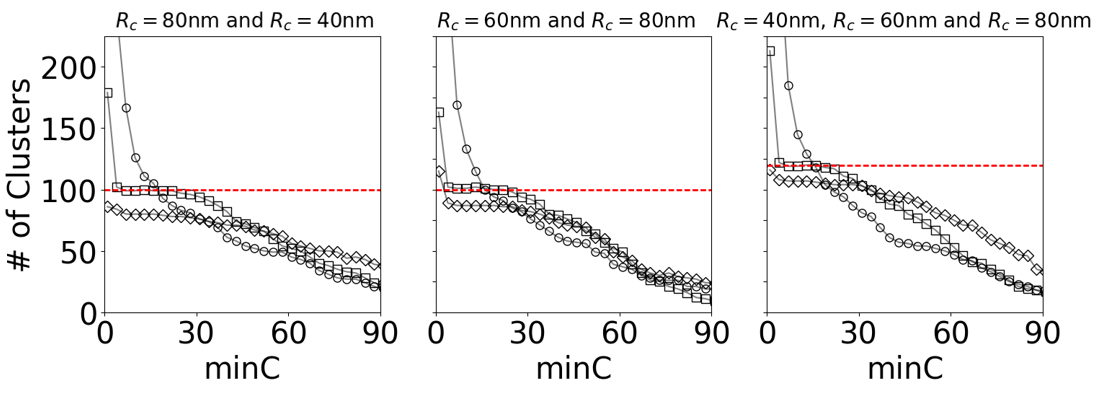

Supplement: S7 Fig — FOCAL3D performance for simulated populations of mixed cluster sizes at a noise level of ζ = 0.01. At each radius, the number of clusters as a function of minC is displayed for a range of grid sizes. (Mixture of Rc = 80 nm and Rc = 40 nm: Δ = 20 nm (∘), 40 nm (▫), 80 nm (◊); mixture of Rc = 80 nm and Rc = 60 nm: Δ = 20 nm (∘), 45 nm (▫), 80 nm (◊); mixture of Rc = 80 nm, Rc = 60 nm, and Rc = 40 nm: Δ = 20 nm (∘), 35 nm (▫), 80 nm (◊)). The red, dashed line indicates the actual number of simulated clusters: 100 and 120. (TIF) [file pcbi.1008479.s009.tif]

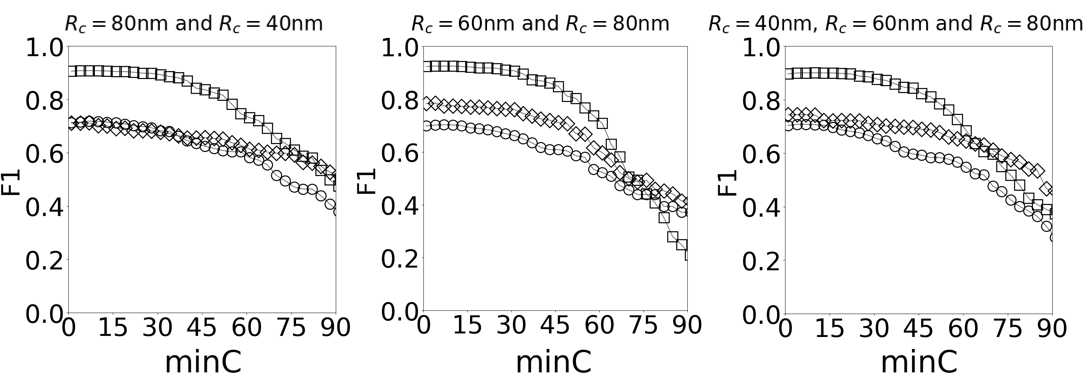

Supplement: S8 Fig — F1 Scores for mixture of Rc = 80 nm and Rc = 40 nm: Δ = 20 nm (∘), 40 nm (▫), 80 nm (◊); mixture of Rc = 80 nm and Rc = 60 nm: Δ = 20 nm (∘), 45 nm (▫), 80 nm (◊); mixture of Rc = 80 nm, Rc = 60 nm, and Rc = 40 nm: Δ = 20 nm (∘), 35 nm (▫), 80 nm (◊). For simulations at ζ = 0.01. (TIF) [file pcbi.1008479.s010.tif]

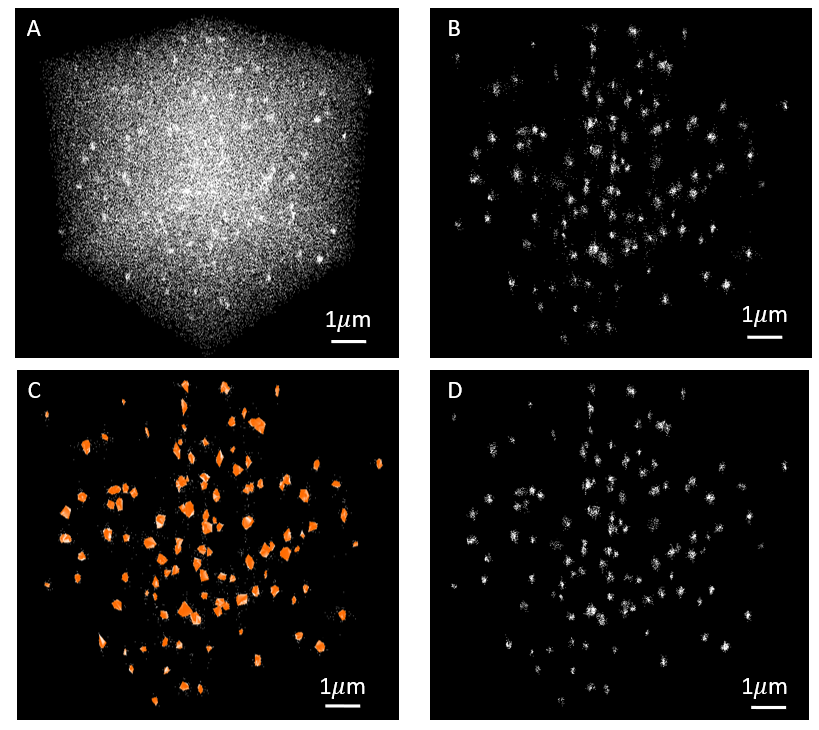

Supplement: S9 Fig — Visualization of the mixed population data set (40 clusters of 40 nm, 60 nm and 80 nm cluster sizes each, for a total of 120 clusters) at moderate noise levels (ζ = 0.01). A. Raw data with noise localizations included. B. Raw cluster data with noise localizations removed (for visualization purposes). C. FOCAL3D clustering results at minC = 13, Δ = 35, as identified from S7 Fig, with noise localizations removed for visualization purposes. D. FOCAL3D results showing only clustered localizations (noise localizations removed for visualization purposes). A few of the localizations at the edge of each cluster are missed, but FOCAL3D precisely and accurately identifies 120 of the clusters (out of 120). (TIF) [file pcbi.1008479.s011.tif]

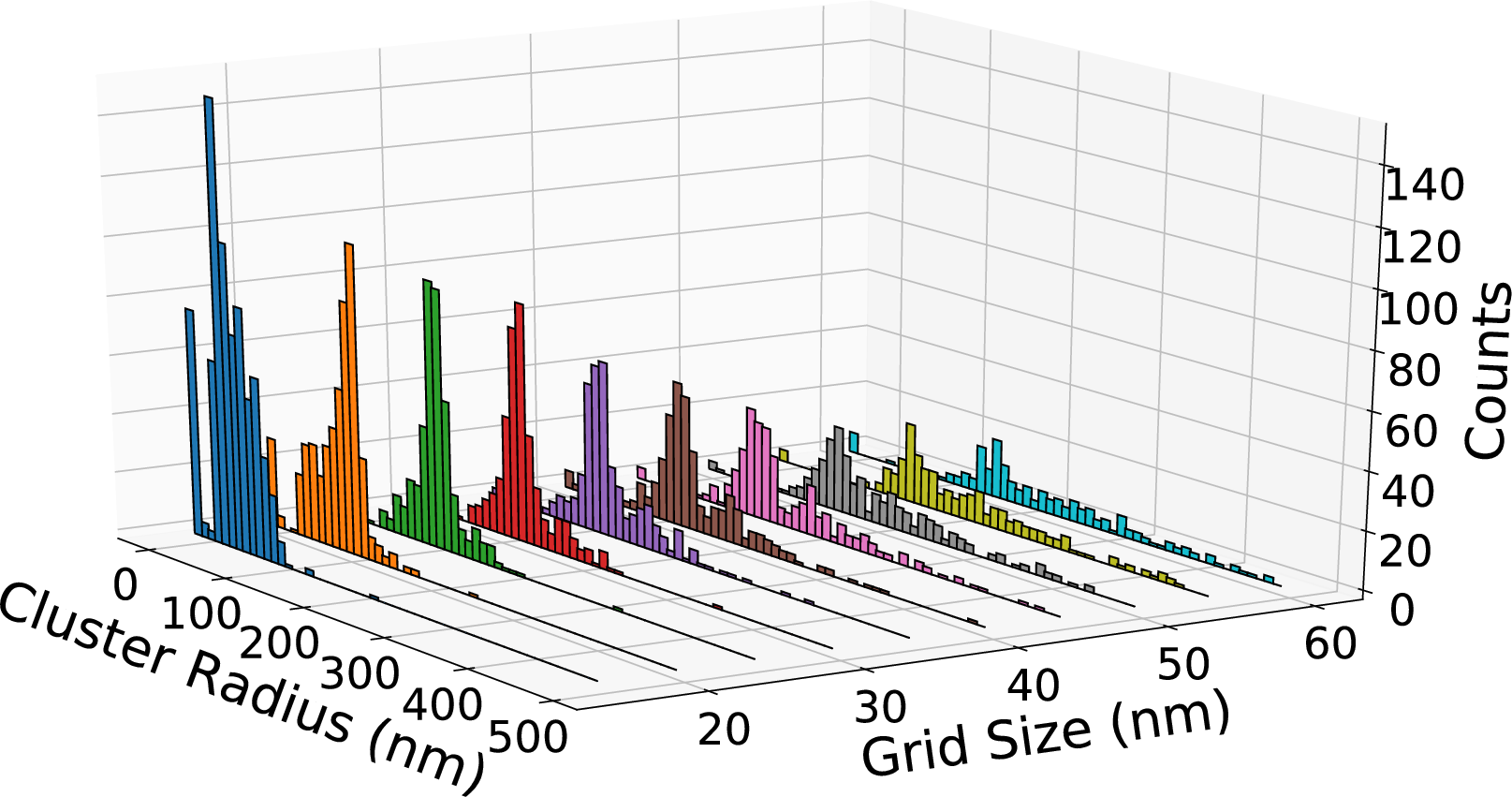

Supplement: S10 Fig — Effective cluster radii for NPC dataset at different grid sizes (evaluated at minC = 46 in Fig 8). For increasing grid size, the distribution in cluster radii first shifts toward a peaked distribution. Then for larger grids, this peaked distribution gradually diminishes while increasingly extending a long tail (indicating large clusters). This is due to separate, but neighbouring, NPCs being grouped into the same cluster. (TIF) [file pcbi.1008479.s012.tif]

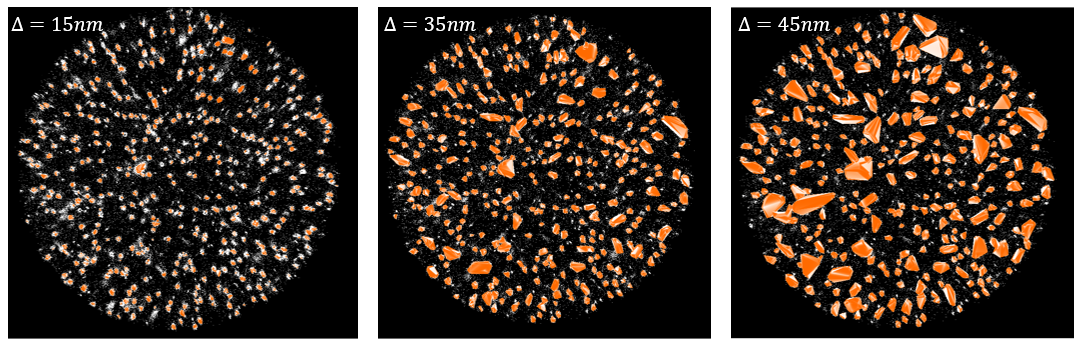

Supplement: S11 Fig — FOCAL3D clustering results at minC = 46 and grid sizes Δ = 15 nm, 35 nm, and 45 nm. The optimal grid size, shown in the main text, was selected to be Δ = 25. For too small of a grid size (Δ = 15 nm), FOCAL3D misses many clusters. For larger grid sizes (Δ = 35 nm and 45 nm), FOCAL3D joins neighbouring distinct clusters together, indicating sub-optimal performance for this data set. (TIF) [file pcbi.1008479.s013.tif]

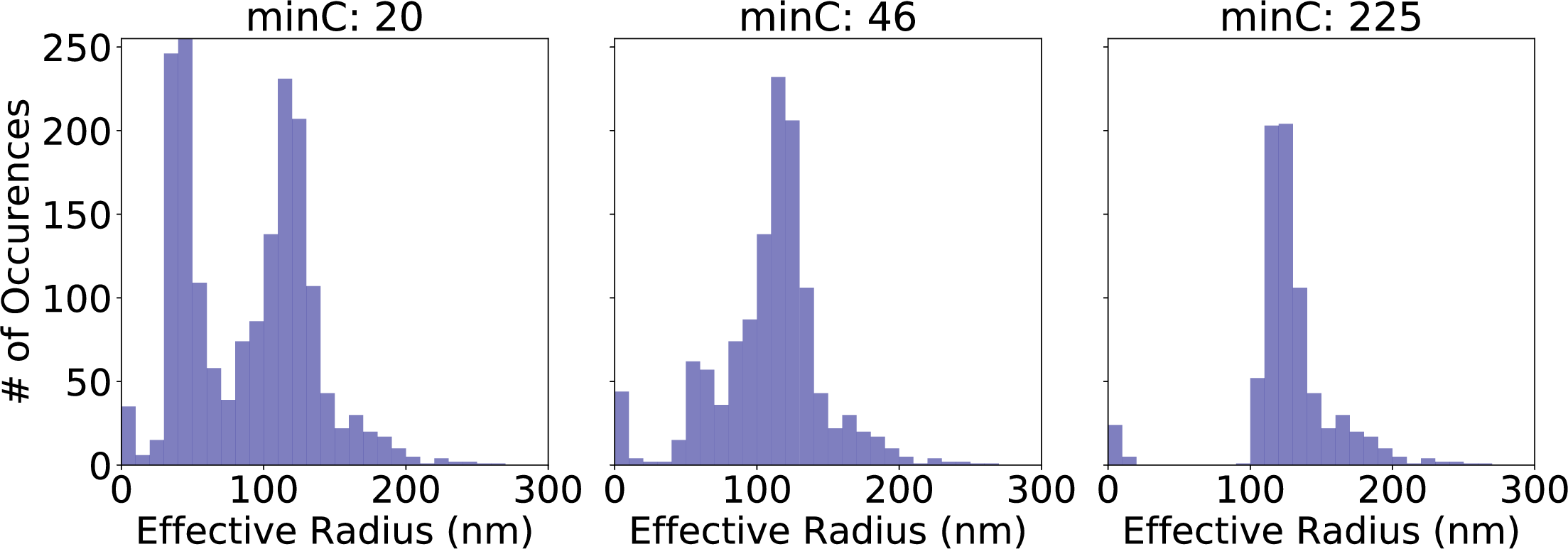

Supplement: S12 Fig — Effective cluster radius, determined by convex hull, for NPC dataset at different values of minC (Δ* = 25 nm). A similar behaviour is observed as in the simulations. For small minC, several false small clusters are identified. About some optimal value, we find a single peaked distribution. And for too large a choice of minC, reasonable, smaller clusters begin to get cut by the size threshold. The average cluster size for a minC of 46 is 109 ± 39. (TIF) [file pcbi.1008479.s014.tif]

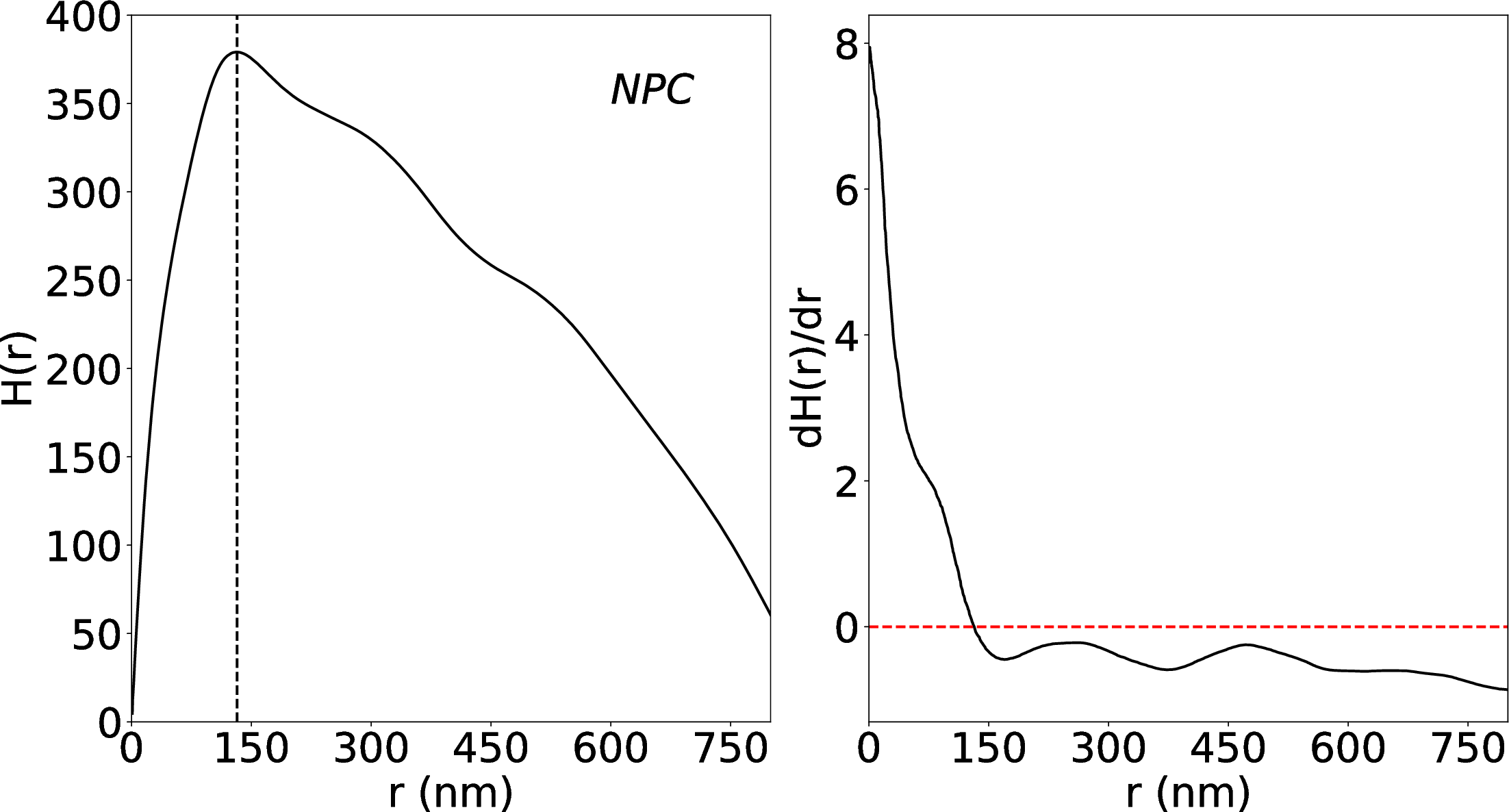

Supplement: S13 Fig — Ripley’s H-function (left) and its derivative (right) for the NPC data set. The cluster size is estimated to be about 132 nm. (TIF) [file pcbi.1008479.s015.tif]
